# Supplementary material for: Predicting perinatal health outcomes using smartphone-based digital phenotyping and machine learning in a prospective Swedish cohort (Mom2B): study protocol
Source: BMJ Open. 2022 Apr 26;12(4):e059033. doi: 10.1136/bmjopen-2021-059033 (PMC9047888; doi:10.1136/bmjopen-2021-059033)
Supplement: Supplementary data [file bmjopen-2021-059033supp001.pdf]

Translated English version of participant information and consent form given digitally to participants via the Mom2B app. See further below for original Swedish version.

PARTICIPANT INFORMATION MOM2B
We want to ask you if you want to participate in a research project. Now you will receive information about the project and what it means to participate. There is a lot of text to read, but the text can also be read again in the app.
At the end of the information, you will have to give consent to the study. You can go into settings at any time and change your consents.
If you have questions about the study, you are welcome to contact us by e-mail: mom2b@kbh.uu.se.

WHAT IS THE PROJECT AND WHY DO YOU WANT ME TO PARTICIPATE?
Postpartum depression is when women experience depression during pregnancy and up to one year after the baby is born. About 12% of all mothers experience postpartum depression. This can lead to suffering and problems for the affected mother and her children. There are effective treatment options. If research finds better ways to identify women at high risk for postpartum depression, more people could receive care early.

During pregnancy and childbirth, the mother and baby can have complications. An example is premature birth, when the baby is born before 37 weeks of pregnancy. Children born prematurely are at greater risk of becoming ill as newborns. In Sweden, about 5% of all births are premature. If the research provides better knowledge to predict which women are at higher risk of being affected, some complications could perhaps be prevented.
With the help of smartphones, it is possible to collect information about people's everyday lives. Examples of measurements are how much a person moves and how active they are on social media. Such measurements have in previous studies been able to predict mental state, but it has not been studied in pregnant women or new mothers. No one has studied whether the measurements can predict complications during pregnancy and childbirth.

THE PURPOSE OF THIS STUDY
The purpose is to use data collected through this app, MOM2B, to improve the detection of women who are at higher risk of developing mental or physical illness during pregnancy and childbirth.

HOW DOES THE STUDY WORK?
You can participate in the study if these three points apply to you:
• I am pregnant or have given birth less than three months ago.
• I am currently using my own smartphone.
• I am at least 18 years old.
At the end of this information section, you can choose which parts of the study you wish to participate in. As a participant, you can choose to contribute:
• To answer questions via this app about your health, lifestyle and background as well as about the child. The questions are compiled by the research team and examine possible risk factors. Questions are sent out approximately 1-2 times per week and usually contain about 5 questions, a maximum of about 15 questions.

• Information about your general mobile use. This means how much internet you use, how much of the mobile processor you use, how often you have access to the internet and how often the charger is connected. The information is collected passively without you having to enter it.
• Information on how much you move. Then the phone's GPS and accelerometer are used. We do not save any specific GPS coordinates, but only information about how you moved in relation to a randomly selected location (eg 100m north, then 50m east, etc.). The information is collected passively without you having to enter it.
• Information about how active you are on social media. What is measured is the number of times someone "likes" something you posted, the number of times you make a status update and the number of times you send a message. Only the number of activities is saved, not the content. What you like or what text you wrote to whom is not saved. The information is collected passively without you having to enter it. The social media that is included right now is Facebook. There may be more (such as Instagram, LinkedIn, Twitter and Snapchat) but then you will get a new question about participation for these.
• To record the sound of your voice when reading texts or numbers. With the help of these audio recordings, we examine e.g. speech tempo and voice mode.
• Information from national health registers and care quality registers about your health, lifestyle and background from one year before the birth to 15 years after the birth. Examples of information that can be obtained are diagnoses you receive in contact with healthcare, level of education, income and employment. The information is collected via the National Board of Health and Welfare and Statistics Sweden.

POSSIBLE CONSEQUENCES AND RISKS OF PARTICIPATING IN THIS STUDY
Answering questions about mental health can involve a risk of emotional strain as some questions may be perceived as sensitive. You choose which questions you want to answer and if you need support, you can contact us for guidance.
If your questionnaire responses indicate severe symptoms of depression, you will receive an automatic message with information on how to seek care. Because your answers are coded and encrypted, we cannot contact you through the app, but you will have the opportunity to enter your phone number so that we can call you during office hours.
Although the research group takes measures to achieve very high data security (such as storing data under code instead of identity, encrypting data directly when collecting and storing data on Uppsala University's servers with a high level of physical and electronic security), it is impossible to completely rule out data intrusion. If data breach is suspected, it will be handled in accordance with current legislation.

ARE THERE ANY BENEFITS?
As a participant, you get access to a knowledge bank in the MOM2B app about health during pregnancy and after childbirth. You also get the opportunity to submit proposals for new topics to the knowledge bank. In addition, participation will not provide any immediate benefits.

WHAT HAPPENS TO MY TASKS?
The project will collect and register information about you. Here we describe how we protect your information.

Code number
You will automatically receive a code number so that no information is stored together with your identity. When we analyze the data and report the results, you will remain anonymous. Responses to questionnaires will be presented so that individuals' responses cannot be traced. The link between your code number and your identity is stored in an isolated location, separate from the information collected.

Encryption
Immediately when data is collected, an encryption is performed. This means that the information is made illegible. Only with the encryption key will the information be readable. All transport of data over networks is done with an additional encryption. The encryption key is stored on a storage unit without a network connection, which is stored locked in a safe at Uppsala University.

Storage of data
The collected information is stored coded and encrypted on a server at Uppsala University indefinitely. Prior to our analyses, the encryption will be unlocked, but the information will remain coded. Only researchers in our research group will have access to the data. Our main analysis, so-called machine learning, is done on computers in a locked space at Uppsala University that has no network connection. All in all, this means that the risk of data intrusion is greatly minimized.
You can choose whether some of your survey answers are to be presented in simplified form on your smartphone under "Statistics". No other data is stored on the phone itself. Identifiable personal data will never be shared with any third party other than the administrators at the National Board of Health and Welfare, Statistics Sweden and the quality of care register, who retrieve information from health registers for the participants who have consented to this. Immediately after the link, all data is deidentified again by coding.
Your answers and your results will be processed so that unauthorized persons cannot take part in them. Uppsala University is responsible for your personal data. According to the EU Data Protection Regulation, you have the right to access the information about you that is handled in the study free of charge and, if necessary, have any errors corrected. You can also request that information about you be deleted and that the processing of your personal data be restricted. If you want to take part in the information, you should contact the principal researcher, Alkistis Skalkidou, telephone: 070 167 97 78 or e-mail: mom2b@kbh.uu.se. The Data Protection Officer can be reached by e-mail: dataskyddsombud@uu.se. If you are dissatisfied with how your personal data is processed, you have the right to report this to the Swedish Data Inspectorate, which is the supervisory authority. The answers to surveys will be compiled statistically in anonymous form and presented so that individuals' answers cannot be traced.

How do I get information about the results of the study?
Results from the project will be presented on our website https://www.mom2b.se/, in scientific articles and at national and international conferences. The results of the study will present the participants' answers in groups and your individual answers will not be traceable to you.

Insurance and compensation
As a participant in this study, you are covered by the patient injury association. No compensation will be given for participating in the study.

PARTICIPATION IS VOLUNTARY
Your participation is voluntary and you can choose to cancel your participation at any time. If you choose not to participate or want to cancel your participation, you do not have to state why, nor will it affect your future care or treatment.
If you would like to cancel your participation, please contact the lead researcher, Alkistis Skalkidou (see below). You are also welcome to contact us at mom2b@kbh.uu.se if you have questions about the study.

Responsible for the study are:
Professor Alkistis Skalkidou
Department of Women's and Children's Health
Akademiska Sjukhuset, 751 85 Uppsala

Email: mom2b@kbh.uu.se
Phone: 070 167 97 78

STEP 1: CONSENT
I have received written information about the study and have had the opportunity to ask questions. I have access to the written participant information electronically in the MOM2B smartphone application at any time.
In the paragraphs that come now, you decide which parts of the study you want to participate in by clicking in the boxes for Yes or No.
I approve of my participation in the study. (You must answer ‘Yes’ to be able to participate in the study.)
□ Yes □ No.

STEP 2: SURVEYS
I agree to answer questions about my health, lifestyle and background as well as the child's health.
□ Yes □ No.

STEP 3: MOBILE USE
I agree that data about my general mobile use is stored. This means internet consumption (kb/s), processor usage (CPU%/s), internet access and whether my phone is charging or not.
□ Yes □ No.

STEP 4: MOBILITY PATTERN
I agree that data is stored about how much I move. We want to record how much and how fast you move, but not exactly where. Only location data from an unknown point is saved, not your exact location. If you select "no", no location data, relative or exact, is saved.
□ Yes □ No.

STEP 5: SOCIAL MEDIA

I agree that information about how active I am on social media is stored. This means the number of times I perform an activity (that someone likes one of your status updates or that you post a status update) but not the text or image content of the activity. Therefore, this does NOT mean information about who is contacted, what is written or what is liked.
□ Yes □ No.

STEP 6: AUDIO RECORDING
I agree to record the sound of my voice when I read out predetermined texts and numbers.
□ Yes □ No.

STEP 7: HEALTH REGISTERS
I agree that information is obtained from national health registers and care quality registers about my health, lifestyle and background from one year before to 15 years after the birth. Examples are diagnoses I get in contact with healthcare, level of education, income and employment.
□ Yes □ No.

STEP 8: PARTICIPATION IN STUDIES
To improve the app and increase the possibilities for predicting postpartum depression and facilitate other studies in women's health, we would like to ask you if we can contact you at a later opportunity with a request for participation in other studies.
At that time, you always have the opportunity to decline the offer of participation. Participation in other studies is completely voluntary and if you choose not to be contacted, you will not be negatively affected in any way. Select one or both of the following options to be contacted:
□ I wish to be contacted for inquiries about participation in sub-studies within Mom2B
□ I wish to be contacted for inquiries about participation in other relevant studies on women's health

SIGNATURE
To be able to use the app, we need your signature
First and last name
Click here to sign

Original Swedish version of participant information and consent form given digitally to participants via the Mom2B app.

DELTAGARINFORMATION MOM2B
Vi vill fråga dig om du vill delta i ett forskningsprojekt. Nu kommer du att få information om projektet och vad det innebär att delta. Det är mycket text att läsa men texten finns också att läsa igen i appen.
I slutet av informationen kommer du få lämna samtycke till studien. Du kan när som helst gå in i inställningar och ändra dina samtycken.
Om du har frågor om studien är du välkommen att kontakta oss på e post: mom2b@kbh.uu.se.

VAD ÄR DET FÖR PROJEKT OCH VARFÖR VILL NI ATT JAG SKA DELTA?
Förlossningsdepression kallas det när kvinnor drabbas av nedstämdhet under graviditeten och upp till ett år efter att barnet fötts. Ungefär 12% av alla mammor får förlossningsdepression. Detta kan leda till lidande och problem för den drabbade mamman och hennes barn. Det finns effektiva behandlingsalternativ. Om forskningen hittar bättre sätt att identifiera kvinnor med hög risk för förlossningsdepression skulle fler kunna få vård tidigt.
Under graviditeten och vid förlossningen kan mamman och barnet råka ut för komplikationer. Ett exempel är för tidig födsel, när barnet föds innan 37 graviditetsveckor. Barn som föds för tidigt har större risk för att bli sjuka som nyfödda. I Sverige är cirka 5% av alla födslar för tidiga. Om forskningen ger bättre kunskap för att förutsäga vilka kvinnor som har högre risk att drabbas skulle en del komplikationer kanske kunna förhindras.
Med hjälp av smartphones går det att samla in information om människors vardagsliv. Exempel på mätningar är hur mycket en person förflyttar sig och hur aktiv den är på sociala medier. Sådana mätningar har i tidigare studier kunnat förutsäga psykiskt mående, men det har inte studerats hos gravida kvinnor eller nyblivna mammor. Ingen har heller studerat om mätningarna kan förutsäga komplikationer under graviditeten och vid förlossningen.

SYFTET MED STUDIEN
Syftet är att använda data som samlas via den här appen, MOM2B, för att förbättra upptäckandet av kvinnor som har en högre risk att drabbas av psykisk eller fysisk sjuklighet under graviditet och förlossning.

HUR GÅR STUDIEN TILL?
Du kan delta i studien om dessa tre punkter stämmer in på dig:
- Jag är gravid eller har fött barn för mindre än tre månader sedan.
- Jag använder just nu min egen smartphone.
- Jag är minst 18 år gammal.

I slutet av det här informationsavsnittet kan du välja vilka delar av studien du önskar delta i. Som deltagare kan du välja att bidra med:
- Att svara på frågor via den här appen om din hälsa, livsstil och bakgrund samt om barnet. Frågorna är sammanställda av forskarteamet och undersöker möjliga riskfaktorer. Frågor skickas ut ungefär 1-2 gånger per vecka och innehåller som regel ca 5 frågor, max ca 15 frågor.

- Uppgifter om din allmänna mobilanvändning. Med detta menas hur mycket internet du använder, hur mycket av mobilens processor du använder, hur ofta du har tillgång till internet och hur ofta laddaren är inkopplad. Informationen samlas passivt utan att du behöver skriva in den.
- Uppgifter om hur mycket du förflyttar dig. Då används telefonens GPS och accelerometer. Vi sparar inte några specifika GPS-koordinater, utan bara information om hur du rört dig i förhållande till en slumpmässigt utvald plats (t.ex. 100m norrut, sen 50m österut, osv.). Informationen samlas passivt utan att du behöver skriva in den.
- Uppgifter om hur aktiv du är på sociala medier. Det som mäts är antalet gånger någon ”gillar” något du postat, antalet gånger du gör en statusuppdatering och antalet gånger du skickar ett meddelande. Bara antalet aktiviteter sparas, inte innehållet. Vad du gillar eller vilken text du skrivit till vem sparas inte. Informationen samlas passivt utan att du behöver skriva in den. De sociala medier som ingår just nu är Facebook. Det kan komma att bli fler (så som Instagram, LinkedIn, Twitter och Snapchat) men då kommer du att få en ny fråga om deltagande för dessa.
- Att spela in ljudet av din röst när du läser upp texter eller siffror. Med hjälp av dessa ljudinspelningar undersöker vi t.ex. taltempo och röstläge.
- Uppgifter från nationella hälsoregister och vårdkvalitetsregister om din hälsa, livsstil och bakgrund från ett år före förlossningen till 15 år efter förlossningen. Exempel på information som kan inhämtas är diagnoser du får i kontakt med vården, utbildningsnivå, inkomst och sysselsättning. Uppgifterna hämtas via Socialstyrelsen och Statistiska Centralbyrån.

MÖJLIGA FÖLJDER OCH RISKER MED ATT DELTA I STUDIEN
Att besvara frågor om psykisk hälsa kan innebära en risk för känslomässig påfrestning eftersom vissa frågor kan uppfattas som känsliga. Du väljer själv vilka frågor du vill svara på och ifall du behöver stöd kan du kontakta oss för vägledning.
Om dina enkätsvar skulle tyda på allvarliga symptom på nedstämdhet kommer du att få ett automatiskt meddelande med information om hur du kan söka vård. Eftersom dina svar är kodade och krypterade kan vi inte kontakta dig genom appen, men du kommer att ha möjlighet att ange ditt telefonnummer så att vi kan ringa upp dig under kontorstid.

Trots att forskargruppen vidtar åtgärder för att uppnå mycket hög datasäkerhet (som att lagra data under kod istället för identitet, kryptera data direkt vid insamling och förvara data på Uppsala Universitets servrar med hög nivå av fysisk och elektronisk säkerhet) är det omöjligt att helt utesluta dataintrång. Om dataintrång mot förmodan skulle ske kommer det att hanteras enligt gällande lagstiftning.

FINNS DET NÅGRA FÖRDELAR?

Du som deltagare får tillgång till en kunskapsbank i MOM2B-appen om hälsa under graviditet och efter förlossning. Du får också möjlighet att skicka in förslag på nya ämnen till kunskapsbanken. Utöver detta kommer deltagandet inte ge några omedelbara fördelar.

VAD HÄNDER MED MINA UPPGIFTER?

Projektet kommer att samla in och registrera information om dig. Här beskriver vi hur vi skyddar dina uppgifter.

Kodnummer
Du får automatiskt ett kodnummer så att inga uppgifter lagras tillsammans med din identitet. När vi analyserar uppgifterna och redovisar resultaten kommer du att förbli anonym. Svar på enkäter kommer att presenteras så att enskilda personers svar inte kan spåras. Kopplingen mellan ditt kodnummer och din identitet lagras på en isolerad plats, separat från de insamlade uppgifterna.

Kryptering
Direkt när uppgifter samlas in görs en kryptering. Detta betyder att informationen görs oläslig. Bara med krypteringsnyckeln blir uppgifterna läsliga. Alla transporter av data över nätverk görs med ytterligare en kryptering. Krypteringsnyckeln sparas på en lagringsenhet utan nätverkskoppling, som förvaras inlåst i ett kassaskåp på Uppsala Universitet.

Förvaring av datauppgifter
De insamlade uppgifterna sparas kodade och krypterade på en server vid Uppsala Universitet på obestämd tid. Inför våra analyser kommer krypteringen att låsas upp, men uppgifterna förblir kodade. Bara forskare i vår forskargrupp kommer att ha tillgång till uppgifterna. Vår huvudsakliga analys, s.k. maskininlärning, görs på datorer i ett låst utrymme på Uppsala Universitet som inte har någon nätverksuppkoppling. Detta innebär sammantaget att risken för dataintrång kraftigt minimeras.

Du kan välja ifall vissa av dina enkätsvar ska presenteras i förenklad form på din smartphone under ”Statistik”. Inga andra uppgifter lagras på själva telefonen.

Identifierbara personuppgifter kommer aldrig att delas med någon tredje part förutom de handläggare på Socialstyrelsen, Statistiska Centralbyrån och vårdkvalitetsregister som hämtar information från hälsoregister för de deltagare som samtyckt till detta. Omedelbart efter sammanlänkningen avidentifieras all data igen genom kodning.

Dina svar och dina resultat kommer att behandlas så att inte obehöriga kan ta del av dem. Ansvar för dina personuppgifter har Uppsala Universitet. Enligt EU:s dataskyddsförordning har du rätt att kostnadsfritt få ta del av de uppgifter om dig som hanteras i studien och vid behov få eventuella fel rättade. Du kan också begära att uppgifter om dig raderas samt att behandlingen av dina personuppgifter begränsas. Om du vill ta del av uppgifterna ska du kontakta huvudansvarig forskare, Alkistis Skalkidou, telefon: 070 167 97 78 eller e-post: mom2b@kbh.uu.se. Dataskyddsombud nås på e-post: dataskyddsombud@uu.se. Om du är missnöjd med hur dina personuppgifter behandlas har du rätt att anmäla detta till Datainspektionen, som är tillsynsmyndighet. Svaren på enkäter kommer att sammanställas statistiskt i anonym form och presenteras så att enskilda personers svar inte kan spåras.

Hur får jag information om resultatet av studien?
Resultat från projektet kommer att presenteras på vår hemsida https://www.mom2b.se, i vetenskapliga artiklar och på nationella och internationella konferenser. Studiens resultat

kommer att presentera deltagarnas svar i grupper och dina individuella svar kommer inte att kunna spåras till dig.

Försäkring och ersättning
Som deltagare i denna studie täcks du av patientskadeföreningen. Ingen ersättning kommer att ges för att du deltar i studien.

DELTAGANDET ÄR FRIVILLIGT

Ditt deltagande är frivilligt och du kan när som helst välja att avbryta deltagandet. Om du väljer att inte delta eller vill avbryta ditt deltagande behöver du inte uppge varför, och det kommer inte heller att påverka din framtida vård eller behandling.

Om du vill avbryta ditt deltagande ska du kontakta huvudansvarig forskare, Alkistis Skalkidou (se nedan). Du är också välkommen att kontakta oss på mom2b@kbh.uu.se om du har frågor kring studien.

Ansvarig för studien är:
Professor Alkistis Skalkidou
Institutionen för Kvinnors och Barns hälsa
Akademiska Sjukhuset, 751 85 Uppsala

Epost: mom2b@kbh.uu.se
Telefon: 070 167 97 78

STEG 1: SAMTYCKE
Jag har fått skriftlig information om studien och har haft möjlighet att ställa frågor. Jag har när som helst tillgång till den skriftliga deltagarinformationen elektroniskt i smartphone-applikationen MOM2B.
I de stycken som kommer nu, bestämmer du vilka delar av studien du vill vara med på genom att klicka i rutorna för Ja eller Nej.
Jag godkänner mitt deltagande i studien. (Du måste svara Ja för att kunna vara med i studien.)
□ Ja □ Nej

STEG 2: ENKÄTER
Jag samtycker till att svara på frågor om min hälsa, livsstil och bakgrund samt barnets hälsa.
□ Ja □ Nej

STEG 3: MOBILANVÄNDNING
Jag samtycker till att uppgifter lagras om min allmänna mobil användning. Med detta menas internetförbrukning (kb/s), processoranvändning (CPU%/s), tillgång till internet samt om min telefon är på laddning eller inte.
□ Ja □ Nej

STEG 4: RÖRELSEMÖNSTER

Jag samtycker till att uppgifter lagras om hur mycket jag förflyttar mig. Vi vill registrera hur mycket och hur snabbt du rör dig, men inte exakt var. Bara platsdata från okänd punkt sparas, inte din exakta position. Väljer du "nej" sparas inga platsdata, relativa eller exakta.
□ Ja □ Nej

STEG 5: SOCIALA MEDIER
Jag samtycker till att uppgifter lagras om hur aktiv jag är på sociala medier. Med detta menas antal gånger jag utför en aktivitet (att någon gillar en av dina statusuppdateringar eller att du postar en statusuppdatering) men inte aktivitetens text- eller bildinnehåll. Detta innebär alltså INTE information om vem som kontaktas, vad som skrivs eller vad som gillas.
□ Ja □ Nej

STEG 6: LJUDINSPELNING
Jag samtycker till att spela in ljudet av min röst när jag läser upp förutbestämda texter och siffror.
□ Ja □ Nej

STEG 7: HÄLSOREGISTER
Jag samtycker till att uppgifter hämtas från nationella hälsoregister och vårdkvalitetsregister om min hälsa, livsstil och bakgrund från ett år före till 15 år efter förlossningen. Exempel är diagnoser jag får i kontakt med vården, utbildningsnivå, inkomst och sysselsättning.
□ Ja □ Nej

STEG 8: DELTAGANDE I STUDIER
För att förbättra appen och öka möjligheterna till prediktion av förlossningsdepression samt underlätta för andra studier inom kvinnohälsa, önskar vi fråga dig om vi får kontakta dig vid ett eventuellt senare tillfälle med förfrågan om deltagande i andra studier.
Vid den tidpunkten har du alltid möjlighet att avböja erbjudandet om deltagande.
Deltagande i övriga studier är helt frivilligt och väljer du att inte bli kontaktad kommer du inte att påverkas negativt på något sätt. Markera ett eller båda av följande alternativ om du vill bli kontaktad:
□ Jag önskar bli kontaktad för förfrågan om deltagande i sub-studier inom Mom2B
□ Jag önskar bli kontaktad för förfrågan om deltagande i andra. relevanta studier om kvinnohälsa

UNDERSKRIFT
För att kunna använda appen behöver vi din underskrift
För- och efternamn
Tryck här för att skriva under
