# Supplementary material for: Predicting perinatal health outcomes using smartphone-based digital phenotyping and machine learning in a prospective Swedish cohort (Mom2B): study protocol
Source: BMJ Open. 2022 Apr 26;12(4):e059033. doi: 10.1136/bmjopen-2021-059033 (PMC9047888; doi:10.1136/bmjopen-2021-059033)
Supplement: Author's manuscript [file bmjopen-2021-059033.draft_revisions.pdf]

BMJ Open is committed to open peer review. As part of this commitment we make the peer review history of every article we publish publicly available.

When an article is published we post the peer reviewers' comments and the authors' responses online. We also post the versions of the paper that were used during peer review. These are the versions that the peer review comments apply to.

The versions of the paper that follow are the versions that were submitted during the peer review process. They are not the versions of record or the final published versions. They should not be cited or distributed as the published version of this manuscript.

BMJ Open is an open access journal and the full, final, typeset and author-corrected version of record of the manuscript is available on our site with no access controls, subscription charges or pay-per-view fees (http://bmjopen.bmj.com).

If you have any questions on BMJ Open's open peer review process please email info.bmjopen@bmj.com

BMJ Open

Predicting perinatal health outcomes using smartphone-based digital phenotyping and machine learning in a prospective Swedish cohort (Mom2B): study protocol

Journal:	*BMJ Open*
Manuscript ID	bmjopen-2021-059033
Article Type:	Protocol
Date Submitted by the Author:	12-Nov-2021
Complete List of Authors:	Bilal, Ayesha; Uppsala University, Department of Neuroscience; Uppsala Universitet, Centre for Women's Mental Health during the Reproductive Lifespan (Womher)<br>Fransson, Emma; Uppsala University, Department of Women's and Children's health; Karolinska Institute, Centre for Translational Microbiome Research, Department of Microbiology, Tumor and Cell Biology<br>Bränn, Emma; Uppsala University, Department of Women's and Children's Health<br>Eriksson, Allison; Uppsala University, Department of Women's and Children's health; Uppsala University, Centre for Women's Mental Health during the Reproductive Lifespan (Womher)<br>Zhong, Mengyu; Uppsala University, Department of Information Technology; Uppsala Universitet, Centre for Women's Mental Health during the Reproductive Lifespan (Womher)<br>Gidén, Karin; Uppsala University, Department of Women's and Children's health<br>Elofsson, Ulf; Uppsala University, Department of Neuroscience, Psychiatry; Uppsala University, Department of Women's and Children's health<br>Axfors, Cathrine; Uppsala Universitet, Department of Women's and Children's Health,<br>Skalkidou, Alkistis; Uppsala Universitet, Department of Women's and Children's health<br>Papadopoulos, Fotios C.; Uppsala Universitet, Department of Neuroscience
Keywords:	Depression & mood disorders < PSYCHIATRY, MENTAL HEALTH, Maternal medicine < OBSTETRICS, PERINATOLOGY, PREVENTIVE MEDICINE, Anxiety disorders < PSYCHIATRY

SCHOLARONE™
Manuscripts

Predicting perinatal health outcomes using smartphone-based digital phenotyping and machine learning in a prospective Swedish cohort (Mom2B): study protocol

Ayesha M Bilal[1, 2], Emma Fransson[3, 4], Emma Bränn[3], Allison Eriksson[2, 3], Mengyu Zhong[2, 5], Karin Gidén[3], Ulf Elofsson[1, 3], Cathrine Axfors[3], Alkistis Skalkidou[3*], Fotios C Papadopoulos[1*]

1. Department of Neuroscience, Psychiatry, Uppsala University, Uppsala, Sweden
2. Centre for Women's Mental Health during the Reproductive Lifespan (Womher), Uppsala University, Uppsala, Sweden.
3. Department of Women's and Children's health, Uppsala University, Uppsala, Sweden
4. Centre for Translational Microbiome Research, Department of Microbiology, Tumor and Cell Biology, Karolinska Institutet, Stockholm, Sweden
5. Department of Information Technology, Uppsala University, Uppsala, Sweden

*equal contribution

Correspondence: Ayesha Mae Bilal
Akademiska sjukhuset 751 85 Uppsala
Email: ayesha.bilal@neuro.uu.se

Word count: 3,868

For peer review only

ABSTRACT

Introduction: Complications in the peripartum period, such as perinatal depression and preterm birth, are major causes of morbidity and mortality for the mother and the child. Prediction of women at high risk is key to minimizing the impact of peripartum complications, by early delivery of existing interventions for prevention. This ongoing study aims to use digital phenotyping data from the Mom2B smartphone application to develop models to predict women at high risk for mental and somatic complications.

Methods and analysis: The Mom2B smartphone app launched in November 2019, and all Swedish-speaking women over 18 years, who are either pregnant or within three months postpartum are eligible to participate. Throughout the pregnancy and within the first year postpartum, both active and passive data is collected via the Mom2B app in an effort to establish a participant's digital phenotype. Active data collection consists of weekly surveys related to participant background information, mental and physical health, lifestyle, and social circumstances, as well as voice recordings. Participants' general smartphone activity, geographical movement patterns, social media activity, and cognitive patterns can be estimated through passive data collection from smartphone sensors and activity logs. The outcomes will be assessed by administering surveys, such as the Edinburgh Postnatal Depression Scale (EPDS), and through linkage to national registers, from where information on registered clinical diagnoses and received care, including prescribed medication, can be obtained.

Advanced machine learning and deep learning techniques will be applied to these multimodal data in order to develop accurate algorithms for the prediction of perinatal depression and preterm birth. In this way, earlier intervention will be possible.

Ethics and dissemination: Ethical permission has been obtained for the Mom2B study and the project fully fulfills the GDPR requirements. Results from this project will be disseminated in international peer-reviewed journals and presented in relevant conferences.

Keywords: perinatal depression, preterm birth, prediction, digital phenotyping, smartphone application, machine learning, deep learning

STRENGTHS AND LIMITATIONS OF THIS STUDY

1. The study collects large-scale, temporally sensitive data regarding the user's behaviors in the real world.
2. End-users' feedback collected allows for app updates and improvements.
3. Passive data collection is expected to have lower attrition rate.
4. Active data collection is prone to suffer from a higher attrition rate.
5. High costs of recruiting participants, and maintaining front- and backend for the smartphone app.

For peer review only - http://bmjopen.bmj.com/site/about/guidelines.xhtml

INTRODUCTION

Optimal maternal health is important throughout pregnancy, childbirth and the postnatal period, to ensure the full potential for the mother, infant and family to get a good start[1]. Two health conditions that are important to address in order to reach a goal of good maternal health is perinatal depression (PND) and preterm birth (PTB), both affecting about every tenth pregnancy worldwide[2, 3].

Perinatal depression

PND is an episode of major depression with onset anytime during pregnancy and up to four weeks postpartum[4], although in research settings, a period of up to one year postpartum is often considered[5]. Antenatal depression (AND) affects between 7-13% of pregnant women[6], and postpartum depression (PPD) is estimated to affect between 10-20% of all newly-delivered mothers[2], while many women experience persistent depression throughout the perinatal period[7]. PND is distinct from "baby blues", which are commonly experienced symptoms of low mood and anxiety that subside within two weeks postpartum. PND is both emotionally and physically debilitating like major depression, with additional risks related to the pregnancy and birth, such as PTB, low birth weight, preeclampsia and placental abnormalities[8-10]. Moreover, it is associated with retained maternal weight postpartum[11], decreased breastfeeding[12-14], poor maternal sleep[15], and poor perinatal quality of life[16]. PND can also compromise the critical mother-infant bond, as it affects the mother's caregiving abilities and adaptation to the maternal role[16, 17], and has a long-term impact on the child's cognitive, emotional and behavioral development[18, 19]. Furthermore, PND can be characterized by the occurrence of self-harm thoughts, which are linked to long-term somatic and psychiatric morbidity[20], and increased maternal mortality from suicide in the first year postpartum[21].

The etiology of PND is multifactorial, including biological, genetic, psychological, and social factors, such as stressful life events, social support, domestic violence, childhood adversity, history of depression and anxiety, low self-esteem, and even personality traits like resilience[15, 22-24]. Despite this knowledge, detecting PND remains a challenge for the healthcare system, with one review finding that around 30-70% of cases go undetected and only 15% receive adequate treatment[25, 26].

Current screening protocols include the Edinburgh Postnatal Depression Scale (EPDS)[27] during postnatal visits to assess risk of PND[28]. However, early detection of PND has remained challenging for many reasons, including inconsistencies in screening practices[29], and failure to distinguish depressive symptoms due to their overlap with typical somatic experiences of early postpartum period[30]. Furthermore, women may hesitate to seek care, possibly because of the depression itself, but also stigma and a fear of being judged as an imperfect mother, as well as concerns about antidepressant use during pregnancy and breastfeeding[31]. Besides, in-clinic screening frequently relies on retrospective self-reports of diagnostically relevant information, making it susceptible to errors and biases associated with autobiographical recollection[32].

Unquestionably, more efficient and effective methods for predicting PND in mothers are needed to enable early identification and intervention, thus improving prognosis, and reducing the burden of disease[33]. Previous studies that have attempted to develop predictive models of maternal depression primarily focus on the postpartum period only[34-39]. Few have used social media fingerprints[40-42] or biomarkers[36, 43] in their models, and these studies largely depended on psychometric self-reports and limited modalities. These

For peer review only - http://bmjopen.bmj.com/site/about/guidelines.xhtml

drawbacks compromise the predictive power of the models and illustrate why multivariate, real-time and unobtrusive approaches to data collection and symptom-monitoring must be encouraged to develop better predictive models.

The primary aim of this study is to use multimodal digital phenotyping data collected through the Mom2B smartphone app to predict the development of PND by implementing advanced machine learning (ML) and deep learning (DL) techniques on large-scale data.

Preterm birth

Among somatic pregnancy complications, PTB is a major cause of neonatal death, as well as of poor long-term health in children, affecting approximately 15 million babies worldwide each year[44, 45]. In Sweden, the preterm birth rate is about 6%[46], which is a relatively low number compared to the international average of over 10%[44]. Like PND, the etiology of PTB is multifactorial, including previous PTB, multifetal pregnancy, cervical insufficiency, intrauterine infections, vaginal bleeding in the second trimester, in vitro fertilization, primiparity, as well as maternal antenatal stress and depression[47-50]. In fact, many risk factors overlap between PND and PTB, such as childhood traumatic events or maltreatment, stressful life events, being single or lacking social support, being overweight, smoking, and low socioeconomic status[51, 52]. Inflammation has been suggested as a possible underlying pathway for both depression and preterm delivery[53].

There are evidence-based interventions for preventing or delaying PTB to optimize birth outcomes, such as smoking cessation, progesterone therapy, cerclage in women with cervical insufficiency, or antibiotics[51, 54]. However, a major obstacle for the success of these interventions is the etiological heterogeneity of PTB, which makes it extremely challenging to identify women at high risk. In fact, two-thirds of women who experience PTB do not present with any risk factors at all[55]. Available biological diagnostic tests for PTB (such as fetal fibronectin) have not sufficient positive prediction values[56]. Screening for cervical length is performed in Sweden for women with a history of previous PTB, however, this is not helpful in primiparous women[57].

It can be concluded that no single biomarker is sufficient for prediction, and multimodal data, including psychosocial and behavioral factors, should be the focus of prediction efforts. To that end, a secondary aim of this study is to use multimodal digital phenotyping data, together with health and pregnancy information from national registers, and utilize advanced ML to predict the risk of PTB.

Digital phenotyping and big data

Digital devices like smartphones allow us to capture moment-by-moment, objective data regarding the patient's experiences and functions in non-clinical settings. This process, known as *digital phenotyping*[58], allows us to collect two kinds of data: *active data* and *passive data*. Active data refers to data that requires user input, such as surveys and voice recordings. Passive data refers to automatically collected data from smartphone sensors and activity logs, which can be used to infer the user's mobility and sleep patterns, digital social activity, smartphone usage patterns, and even affective and cognitive changes.

The Mom2B smartphone app is developed using the Beiwe research platform (www.beiwe.org) from the Harvard School of Public Health. It can allow us to capture digital phenotyping data during the perinatal period with greater efficiency and temporal sensitivity as data collection occurs continuously and in real-world contexts, which minimizes the risk of recall biases. Such apps could also be integrated into the mother's perinatal care plan. One

For peer review only - http://bmjopen.bmj.com/site/about/guidelines.xhtml

drawback of smartphone-based data collection, in general, is the attrition rate that increases with longer follow-up times[59], however, this can at least in part be compensated for by the continuous collection of passive data.

In fact, one of the biggest advantages of smartphone-based digital phenotyping is the ability to collect multivariate, high-volume data, known commonly as big data[60]. Big data is excellent for healthcare research since it can facilitate a unique insight into risk factors and the development of better diagnostic frameworks[61], however, the literature on big data approaches for psychiatric conditions, particularly perinatal mental health, is limited[62]. Nordic countries are in the forefront in this respect – with the Danish National Birth Cohort (DNBC)[63], the Norwegian MoBa[64] and ABC[65] studies and the Swedish BASIC study[66] - due to the availability of nationwide registers with comprehensive personal and medical information for all pregnant women in these countries. Nonetheless, register data, while valuable, lacks the multeity, continuity and veracity that digital phenotyping offers[67]. Furthermore, studies derived from these cohorts have largely relied on traditional statistical methods, which are limited in their ability to scale to large datasets and identify more subtle patterns in data[68].

To date, few studies have applied digital phenotyping for prediction of psychiatric conditions, such as relapse in schizophrenia[69] and severity of mood episodes in bipolar disorder[70]. In the context of PND, while smartphone apps are widely used, their application has been largely focused on screening and intervention[71]. Only two studies have applied digital phenotyping for predicting PPD[34, 72]. While these studies have reported encouraging results, their predictive ability is compromised due to limited modalities (using only active data in the form of questionnaires), infrequent measurement points, and usage of more traditional statistical methods. The Mom2B study combines nationwide health and pregnancy register data with active and passive data collected through smartphone-based digital phenotyping to objectively monitor indicators of PND in non-clinical contexts.

In order to harness the full potential of big data, more advanced analytical methods, such as ML and DL, are ideal. ML is an artificial intelligence approach that refers to various methods of enabling an algorithm to identify and learn intricate patterns in data to predict outcomes[73]. Modern ML methods such as deep neural networks (DNNs) are uniquely suited to analyzing big datasets as they can detect complex, high-dimensional interactions and structured information, without guidance, that can then be used to train predictive algorithms. DNN models comprise of multiple “hidden” processing layers, inspired by biological neural networks, consisting of a series of interconnected nodes that resemble neurons[74, 75].

Over the last decade, there has been a steady increase in the use of DL methods in medicine[76]. However, few studies have used ML for diagnosis or risk assessment in psychiatry, and those that do often struggle with limited sample sizes and modalities, or using only traditional ML techniques[73, 77]. To our knowledge, Mom2B is the first study to adopt a big data approach and use multimodal digital phenotyping with advanced ML techniques to develop predictive algorithms for PND and preterm birth.

COHORT DESCRIPTION

Mom2B (www.mom2b.se) is a national ongoing smartphone app-based study; the app was launched at the end of November 2019 to App Store and Google Play. All Swedish-speaking women above the age of 18 owning a smartphone, who are either pregnant or within three months postpartum, are eligible to participate by registering and providing consent in the

For peer review only - http://bmjopen.bmj.com/site/about/guidelines.xhtml

Mom2B app. Participating women are also asked for optional consent to be contacted for other studies that may involve biological sampling or participation in laboratory tests, for example, neurophysiological tests. Participant data is then linked to psychiatric and somatic health- and pregnancy-related information available from Swedish national registers.

We anticipate to recruit 20,000 participants. Due to the complexity of ML methods, it is not possible to perform any traditional test of statistical power. However, based on previous studies[59, 78, 79] and conferring with experts in artificial intelligence, we estimated that this approximate number would give us enough material to build robust prediction models while accounting for attrition and the prevalence rates of the outcomes. Moreover, analyses done recently in the BASIC cohort[39] have led us to consider the sufficiency of much fewer participants for analysis to commence.

Information about the study is being disseminated on social media, as well as through posters and brochures sent to primary and maternal care centers around the country. Figure 1 illustrates Mom2B participant recruitment and data contribution, including consent and opt-out rates. Table 1 outlines the sociodemographic characteristics, pregnancy history and birth outcomes of the existing Mom2B cohort based on users who have contributed relevant data, along with similar characteristics in the general Swedish population.

Characteristics	Mom2B (2020-2021) (n=3166)[a]			Sweden (2019)[b]	
	Available data (*n*)	Missing data (*n*)	n(%) or mean±sd	Available data (*n*)	n(%) or mean
**Maternal age (years)**	2793	373	32.5±4.4	113 816	30.7
**Country of origin**	2803	363		112 530	
Sweden			2583 (92)		78 033 (69.3)
Nordic countries except Sweden			33 (1)		1280 (1.1)
Europe except Nordic countries			100 (3.5)		9172 (8.2)
Outside Europe			87 (3)		24 045 (21.4)
**Education**	2805	361		107 711	
≤ 12 years			589 (21)		48 793 (45.3)
Post-secondary education			2216 (79)		58 918 (54.7)
**Employment before pregnancy**	1075	2091		113 147	
Working/student/parental leave			1045 (97)		103 967 (91.9)
Unemployed/sick leave			30 (3)		9180 (8.1)
**Smoking 3 months before pregnancy**	2533	633	371 (14.5)	110 991	11 765 (10.6)
**BMI before pregnancy (kg/m²)**	2729	437	25.5±5.3	108 929	
<18,5			60 (2)		2783 (2.5)
18,5 – 25			1474 (54)		59 384 (54.6)
25 - <30			746 (27)		29 636 (27.2)
>30			449 (16.5)		17 126 (15.7)
**Primiparous**	2670	496	756 (28)	113 816	48 473 (42.5)
**Cesarean section**	1102	2064	188 (17)	114 757	20 312 (17.7)
**Preterm delivery (<week 36)**	1295	1871	73(5.6)	116 071	6502 (5.6)

Table 1. *Sociodemographic information on participants in the MOM2B study and the general population of pregnant women in Sweden. Percentages given in relation to available data from women. [a]Data downloaded on September 6, 2021. [b]Data retrieved from the Swedish Medical Birth Register and Swedish National Board of Health and Welfare from 2019.*

DATA COLLECTION

Surveys and Questionnaires

Surveys are distributed via the MOM2B app around 1-2 times per week, with a mean of 5 and maximum of 15 questions per survey. The EPDS[27], a ten-item self-report screening tool with good psychometric properties[80, 81], is used as the primary outcome to assess depressive symptoms throughout the study period. A number of validated instruments are used to assess wellbeing and anxiety, as well as known risk factors, such as perceived stress and personality (Table 2). Furthermore, information pertaining to the participant's background, psychological

For peer review only - http://bmjopen.bmj.com/site/about/guidelines.xhtml

and somatic health, lifestyle, and pregnancy- and birth-related experiences is also collected (Table 3).

Category	Surveys	Pregnancy week			Postpartum week			
		0 - 12	13 - 27	28 - 40	0 - 12	13 - 27	28 - 40	40 - 52
Mental Health	EPDS	✓	✓	✓	+	+	✓	✓
	WHO5	+	+	+	+	+	+	+
	DSM- screening		✓			✓		
	DSM- screening Short		✓	✓	✓	✓	✓	
	HADS			✓	✓			
General Health	IPAQ		✓	✓	✓	✓		
	FSFI	✓		✓	✓			
Stress	PSS		✓	✓	✓		✓	✓
	SLE	✓		✓		✓		
	LITE				✓			
	FOBS		+	✓				
Personality & resilience	Rs-14		✓					
	SOC		✓					
	VPSQ			✓		✓		
	ECR			✓		✓		
Bond	S-MIBS				✓			
	PBQ					✓		
Child	IBQ							✓
	ICQ				✓			

Table 2. *Validated instruments in the Mom2B study, administered during different time windows during pregnancy and postpartum. Note: ✓ = Survey administered once during respective time period; + = Survey administered multiple times during respective time period; EPDS, Edinburgh Postnatal Depression Scale[27]; WHO5,World Health Organization wellbeing index[103]; DSM-screening, Diagnostic and Statistical Manual, 5th edition, criterion for depression; DSM Screening – short is a shortened version of the DSM Screening with selected questions chosen by the research team; HADS, Hospital Anxiety and Depression Scale[104]; IPAQ, International physical activity questionnaire[105]; FSFI, Female sexual function index[106]; PSS, Perceived stress scale[107]; SLE, Stressful life events[108]; LITE, Lifetime Influence of Traumatic Experiences[109]; FOBS, Fear of birth[110]; Rs-14, Resilience scale[111]; SOC, Sense of coherence[112]; VPSQ, Vulnerability personality style questionnaire[113]; ECR, Experience in Close Relationships scale[114]; S-MIBS, Swedish Mother to Infant Bonding Scale[115]; Postpartum Bonding Questionnaire (PBQ)[116]; IBQ, Infant Behavior Questionnaire[117]; ICQ, Infant Characteristics Questionnaire[118].*

For peer review only

For peer review only - http://bmjopen.bmj.com/site/about/guidelines.xhtml

Category	Questionnaires	Baseline	Pregnancy week			Postpartum week			
			0 - 12	13 - 27	28 - 40	0 - 12	13 - 27	28 - 40	40 - 52
Background	Number of children	✓							
	Sociodemographic info	✓							
	Lifestyle before pregnancy	✓							
	Pregnancy planning	✓							
	Previous pregnancies	✓							
Medical history	Previous illness	✓							
	Previous mental illness	✓							
	Multiple gestation			✓					
	Weight		+	+	+	+	+		
	Substance abuse			✓				✓	
	Medications			✓		✓	✓		
	Endometriosis				✓			✓	
	Gynecological health	✓							
Lifestyle and circumstances	Meaning of life			✓			✓		
	Social support			+	✓	✓	✓		
	Diet			✓	✓	✓			
	Valentin			✓					
	Coronavirus pandemic		✓	✓	✓	✓	✓		
Pregnancy	Sleep		+	+	✓	+		✓	
	Pregnancy complications		✓	+	+				
	Views on breastfeeding				✓				
Postpartum	Breastfeeding					+	✓	+	✓
	Parental leave				✓	✓	✓		✓
	Neonatal care					✓			
	Physical problems					✓	+		

Table 3. *Questionnaires in the Mom2B study, administered during different time windows during pregnancy and postpartum, by category of information. Note. ✓ = Survey administered once during respective time period; + = Survey administered multiple times during respective time period; Valentin scale (relationship with your partner)[119]*

Voice recordings

Voice acoustic qualities, such as pitch, speed, timing and timbre, have been used in previous research to successfully distinguish depressed from non-depressed individuals[82-85]. To collect voice data, the Mom2B app sends out a voice recording task asking the participant to record reading simple texts, numerical sequences or vocalizations every 2-4 weeks.

Passive data collection

The Mom2B app regularly collects passive data, which is used to infer the user's mobility, data usage, smartphone phone usage, sleep, and social media usage parameters. Some of these features are collected differently for iOS and Android users. The feature modalities are briefly explained below:

Mobility

Correlations have been demonstrated between a patient's geographical movement patterns and changes in depressive symptoms[86, 87]. 60 seconds of GPS data is continuously collected every 10 minutes. Accelerator data is collected when the motion exceeds a certain threshold; motion activities, including being stationary, walking, running, cycling, and movement in a vehicle are recorded when the state changes. For iOS, we collect device motion to provide more detailed motion sensor data.

Data usage

For peer review only - http://bmjopen.bmj.com/site/about/guidelines.xhtml

Internet usage is the main feature of data usage. Various patterns of internet usage have been identified in relation to depression in different populations[88, 89], but not among women with PND. The Mom2B app records the accumulated upload and download rates together with timestamps. Another feature, reachability, records timestamped smartphone connectivity – whether the phone is connected to cellular network, Wi-Fi or neither. It also records a Wi-Fi log for Android phone, which includes anonymized MAC addresses' frequencies and signal strength (RSSI) of available wireless networks in the area.

Smartphone phone usage

General smartphone use has been found to correlate with sleep quality and depression[90, 91]. Phone power state, combined with mobility parameters, can reflect individual behavior like sleep patterns[92, 93]. To keep track of the use of the smartphone device, data is collected on screen activity, charging status, and device reboot.

Social media

Social media behavior has also been proven useful in detecting mental states. It has also been shown that reduced social activity on Facebook predicted symptoms of PPD[41]. We collect data on users' Facebook activity, including making posts, comments, or likes, together with their timestamps. We only collect the activity, not the content, of the posts or comments.

Survey metadata

App-based surveys make it possible to also collect metadata. This kind of behavioral metadata may contain clinically relevant information related to attention, processing speed and working memory capacity, and even any deterioration of psychiatric symptoms[58, 94]. We collect data on the time a survey was opened, time taken to answer each question and fully complete a survey, as well as any changes made in survey responses.

National Register data

Supplementary information will be accessed via the following Swedish national health and quality registers: the Medical Birth Register, the Pregnancy Register, the National Patient Register, the Prescribed Drug Register, and population censuses from Statistics Sweden. The accessed information includes records of perinatal complications such as PND, PTB, or any other complications considered important risk factors for the study outcomes, such as gestational diabetes, gestational hypertension, preeclampsia, prolonged delivery, severe lacerations, postpartum hemorrhage, induction of delivery, instrumental vaginal delivery, cesarean section, and small for gestational age.

Further, the mother's weight at enrollment in maternity care and at aftercare visits; calculated date of birth from last menstrual period and from ultrasound; and information on previous miscarriages, previous abortions, chronic diseases, fear of childbirth, involuntary infertility, gestational age at enrollment in maternity care, and fetal diagnostics will be obtained from the Medical Birth Register. Retrieved information also includes variables regarding the background, health, and lifestyle of the participant, for validation purposes of our self-report questionnaires, as well as psychiatric and somatic morbidity for up to 15 years after childbirth.

DATA FLOW AND STORAGE

Figure 2 illustrates the data flow and storage process, which is follows:

For peer review only - http://bmjopen.bmj.com/site/about/guidelines.xhtml

1. Participants register to the study via the Mom2B app using their Swedish Social Security Number, which is encrypted in the device using a private key provided by the Beiwe backend server, and replaced by a random, pseudo-anonymized code number.
2. The decryption key, together with the participant consent information and electronic signatures, is stored in a private, write-only server at Uppsala University.
3. The app fetches surveys and voice recording tasks, and uploads data from participants to the backend server, where it is encrypted and sent to MinIO, a secure, cloud-based storage, where another layer of encryption is added. Passive data collected from the phone follows the same path.
4. From MinIO, all data is sent to Bianca, a private, offline server, in both encrypted and decrypted forms for storage and analysis respectively.
5. The app provides weekly reports based on participant activity and fetches health-related information relating to the perinatal period the user is in, as well as Frequently Asked Questions about the study and perinatal health.

METHODS

Preliminary data analysis strategy

We plan to utilize both traditional ML and DL techniques in order to develop accurate predictive algorithms. Using DL to analyze digital phenotyping data for evaluating risk of depression is a relatively novel approach compared to traditional ML models[73]. DL models have been shown to outperform traditional ML in various tasks involving complex datasets[95, 96], and can be combined with traditional ML in multimodal data mining tasks to further improve performance[97].

Mom2B data exists in different modalities, including audio data, sensor data and survey data. To handle this multimodal data, we will extract features for these modalities separately. We will use traditional feature engineering as well as DL techniques for feature extraction[97, 98]. An example of traditional feature engineering utilizes trajectories[87], which use mobility features, such as number of significant places visited, maximum distance, and standard deviation. However, DL can be used for extracted features for many other modalities, such as social media data and audio data, which are not investigated widely in the area.

Our data will be split into a training dataset and a test dataset, and we will develop multi-modal models to comprehensively analyze the data using the training dataset, and assess the performance of models using the test dataset. Thereby, we can compare the performance of traditional ML with DL, and the different assemblies of models, in the prediction of PND.

Computational model

The multimodal data we collect are in different scales, dimensions and formats, which need to be harmonized before prediction[99]. Different models can perform suitably on different modalities. To handle this complexity in multiple data modalities, we consider modality fusion and model selection during the development phase.

One example of a multimodal ML model is shown in Figure 3. The model is designed to detect potential depressive episodes based on multimodal data collected in the Mom2B app. Pre-processed data in three modalities is fed into models and intermediate representations are then fused together and fed in as input features of a classification model.

Patient and public involvement

For peer review only - http://bmjopen.bmj.com/site/about/guidelines.xhtml

A qualitative study is planned for exploring the attitudes and concerns of participating women towards the Mom2B app. Direct contact with end-users and the ability to make changes to the app based on their feedback can enhance user experience and increase engagement. A representative from Mamma till Mamma, a non-profit organization in Sweden focused on perinatal mental wellbeing, serves on our advisory board. The organization has been involved in the piloting of our study and design of questionnaires, and currently support us with recruitment. We plan to involve them in the dissemination of study results as well.

SUB-STUDIES

In addition to predicting PND and PTB, the rich data collected from the Mom2B cohort will also be used to investigate further questions, mainly regarding the health of pregnant and postpartum women. Other planned areas of research are regarding the impact of early mother-infant separation and neonatal intensive care of the baby on the wellbeing of the mother; and sexual function and its potential correlates to depression and anxiety in the peripartum period.

Maternal depression and well-being during the Covid 19 pandemic

In the beginning of 2021, data collected from 1577 participants was used to assess depressive and anxiety symptoms, as well as wellbeing and life changes in pregnant women in Sweden during the COVID19 pandemic (from February 2020-March 2021)[100]. The Mom2B app enabled gathering psychiatric information at a national level during the pandemic, as well as passive data on mobility. Levels of perinatal affective symptoms and low wellbeing were elevated compared to previous years and to months with fewer cases. Similar apps can help healthcare providers and governmental bodies to monitor high-risk groups during crises in real time, as well as to adjust measures and the support offered.

STRENGTHS AND LIMITATIONS

Besides the utility of digital phenotyping in combination with the advanced analytical methods planned to be used, other strengths of the Mom2B study include the involvement of participants. Statistics and reports are sent back to the user based on the data they contribute, allowing them to follow their own mental health and activity as an incentive for continued participation. Participants also have the possibility to contact the research team for support if depressive symptoms, which the participants get notified of, are present.

The involvement of user organizations and an international advisory board further strengthen the study, by increasing the feasibility, the use of state-of-the-art methods and the potential for high acceptance by the end-users, which is especially important for a future integration with the regular clinical practice.

However, there are some limitations, including the availability of the app only in Swedish, which excludes a number of otherwise eligible participants, and the high costs for maintaining the technical infrastructure in the front- and back-end of the app, which require considerable funding. Attrition is also an issue, especially with data that requires active input from users. While we can attempt to combat this by improving the app based on user suggestions and experiences, it is important to consider that attrition might also reflect the worsening of symptoms and be a predictor per se of clinical deterioration. It will be important to distinguish such participants and determine how to use attrition as a predictor variable.

FUTURE PERSPECTIVES

For peer review only - http://bmjopen.bmj.com/site/about/guidelines.xhtml

We are at the beginning of the smartphone-based research era, and future possibilities seem numerous. We intend to develop the Mom2B app in other languages, including English, to expand to a more diverse and wider population. If the app succeeds in developing good predictive models for PND, the research team anticipate that the app could be further developed to include evidence-based interventions interventions[101]. Furthermore, since PND is much less understood in co-parents and improving the other parent's mental wellbeing is conducive to the health of the mother and the children as well[102], the app could be further developed to study paternal PND. The Mom2B research team plans to further adapt the app to other research topics such as teenage and student mental health, and prediction of new episodes or self-harm in major depression.

ETHICS AND DISSEMINATION

Ethical permit has been obtained for the Mom2B study (DNR: 2019-01170; 2020-01824; 2020-06645) and the project fully fulfills the GDPR requirements, including the processing, storage and protection of all data. Results will be continuously disseminated through international peer-reviewed journals, the project's website and social media channels, and presented in national and international conferences. All publications will be open access.

CONTRIBUTORS

AS and FCP were involved in the conception and design of the study, revised the draft critically for intellectual content and approved the final manuscript. EB, EF and CA contributed significantly to the design and launch of the project, and revised the draft critically for intellectual content. AE, EB, UE, MZ and KG contributed to the drafting of the protocol and the continuation of the project. AMB is the coordinator of the study and was primarily responsible for drafting the manuscript and revising it critically for intellectual content.

FUNDING STATEMENT

This work was supported by the Uppsala Region to AS, the Swedish Association of Local Authorities and Regions (SKR) to the department of Obstetrics and Gynecology, Uppsala University Hospital, the Swedish Research Council (Grant number 2020-01965) to AS, as well as the Swedish Brain Foundation and Uppsala University Womher School.

COMPETING INTERESTS

None.

ACKNOWLEDGEMENTS

We would like to extend our gratitude to Oskar Burhman for creating and continuously adjusting the mobile research application Mom2B; to Dr. Masoumeh Rezapour, the previous head of department of Obstetrics and Gynecology at Uppsala University Hospital, for support and guidance in the first steps of this project; to Arvid Marklund and Stavros Iliadis for support at the early stages of the app creation; and to Maria Grandahl, Emilia Biskop, and Lisa Lindgren for their valuable input regarding app content and questionnaires.

REFERENCES FOR TABLES

[103-119]

REFERENCES

For peer review only - http://bmjopen.bmj.com/site/about/guidelines.xhtml

1. WHO. Maternal Health World Health Organization [Available from: https://www.who.int/health-topics/maternal-health accessed November 2021.
2. Woody CA, Ferrari AJ, Siskind DJ, et al. A systematic review and meta-regression of the prevalence and incidence of perinatal depression. *Journal of Affective Disorders* 2017;219:86-92. doi: 10.1016/J.JAD.2017.05.003
3. WHO. Preterm birth World Health Organization2018 [Available from: https://www.who.int/news-room/fact-sheets/detail/preterm-birth.
4. Association AP. Diagnostic and statistical manual of mental disorders. 5th ed. Washington, DC2013.
5. Moraes GPDA, Lorenzo, L., , Pontes GAR, Montenegro MC, et al. Screening and diagnosing postpartum depression: when and how? *Trends in psychiatry and psychotherapy* 2017;39(1):54-61. doi: 10.1590/2237-6089-2016-0034
6. Bennett HA, Einarson A, Taddio A, et al. Prevalence of depression during pregnancy: Systematic review. *Obstetrics and Gynecology* 2004;103(4):698-709. doi: 10.1097/01.AOG.0000116689.75396.5F
7. Wikman A, Axfors C, Iliadis SI, et al. Characteristics of women with different perinatal depression trajectories. *Journal of Neuroscience Research* 2020;98(7):1268-82. doi: 10.1002/JNR.24390
8. Field T, Diego M, Hernandez-Reif M. Prenatal depression effects on the fetus and newborn: a review. *Infant Behavior and Development* 2006;29(3):445-55. doi: 10.1016/J.INFBEH.2006.03.003
9. Grote NK, Bridge JA, Gavin AR, et al. A meta-analysis of depression during pregnancy and the risk of preterm birth, low birth weight, and intrauterine growth restriction. *Arch Gen Psychiatry* 2010;67(10):1012-24. doi: 10.1001/archgenpsychiatry.2010.111 [published Online First: 2010/10/06]
10. Kurki T, Hiilesmaa V, Raitasalo R, et al. Depression and anxiety in early pregnancy and risk for preeclampsia. *Obstet Gynecol* 2000;95(4):487-90. doi: 10.1016/s0029-7844(99)00602-x [published Online First: 2000/03/22]
11. Herring SJ, Rose MZ, Skouteris H, et al. Optimizing weight gain in pregnancy to prevent obesity in women and children. *Diabetes Obes Metab* 2012;14(3):195-203. doi: 10.1111/j.1463-1326.2011.01489.x [published Online First: 2011/08/20]

For peer review only - http://bmjopen.bmj.com/site/about/guidelines.xhtml

12. Cato K, Sylven SM, Lindbäck J, et al. Risk factors for exclusive breastfeeding lasting less than two months—identifying women in need of targeted breastfeeding support. *PloS one* 2017;12(6):e0179402. doi: https://doi.org/10.1371/journal.pone.0179402
13. Dennis CL, McQueen K. The relationship between infant-feeding outcomes and postpartum depression: a qualitative systematic review. *Pediatrics* 2009;123(4):e736-51. doi: 10.1542/peds.2008-1629 [published Online First: 2009/04/02]
14. Figueiredo B, Canario C, Field T. Breastfeeding is negatively affected by prenatal depression and reduces postpartum depression. *Psychol Med* 2014;44(5):927-36. doi: 10.1017/S0033291713001530 [published Online First: 2013/07/05]
15. Field T. Prenatal Depression Risk Factors, Developmental Effects and Interventions: A Review. *Journal of pregnancy and child health* 2017;4(1) doi: 10.4172/2376-127X.1000301
16. Slomian J, Honvo G, Emonts P, et al. Consequences of maternal postpartum depression: A systematic review of maternal and infant outcomes. *https://doiorg/101177/1745506519844044* 2019;15 doi: 10.1177/1745506519844044
17. Brummelte S, Galea LAM. Postpartum depression: Etiology, treatment and consequences for maternal care. *Hormones and Behavior* 2016;77:153-66. doi: 10.1016/J.YHBEH.2015.08.008
18. Goodman JH. Perinatal depression and infant mental health. *Arch Psychiatr Nurs* 2019;33(3):217-24. doi: 10.1016/j.apnu.2019.01.010 [published Online First: 2019/06/23]
19. Kingston D, Tough S, Whitfield H. Prenatal and postpartum maternal psychological distress and infant development: a systematic review. *Child Psychiatry Hum Dev* 2012;43(5):683-714. doi: 10.1007/s10578-012-0291-4 [published Online First: 2012/03/13]
20. Iliadis SI, Skalkidou A, Ranstrand H, et al. Self-harm thoughts postpartum as a marker for long-term morbidity. *Frontiers in public health* 2018;6:34. doi: https://doi.org/10.3389/fpubh.2018.00034

For peer review only

For peer review only - http://bmjopen.bmj.com/site/about/guidelines.xhtml

21. Esscher A, Essen B, Innala E, et al. Suicides during pregnancy and 1 year postpartum in Sweden, 1980-2007. *Br J Psychiatry* 2016;208(5):462-9. doi: 10.1192/bjp.bp.114.161711 [published Online First: 2015/10/24]
22. Lancaster CA, Gold KJ, Flynn HA, et al. Risk factors for depressive symptoms during pregnancy: a systematic review. *Am J Obstet Gynecol* 2010;202(1):5-14. doi: 10.1016/j.ajog.2009.09.007 [published Online First: 2010/01/26]
23. Skalkidou A, Hellgren C, Comasco E, et al. Biological aspects of postpartum depression. *Womens Health (Lond)* 2012;8(6):659-72. doi: 10.2217/whe.12.55 [published Online First: 2012/11/28]
24. O'Hara MW. Postpartum depression: what we know. *Journal of clinical psychology* 2009;65(12):1258-69. doi: https://doi.org/10.1002/jclp.20644
25. Cox EQ, Sowa NA, Meltzer-Brody SE, et al. The perinatal depression treatment cascade: Baby steps toward improving outcomes. 2016;77:1189-200. doi: 10.4088/JCP.15r10174
26. Bränn E, Fransson E, Wikman A, et al. Who do we miss when screening for postpartum depression? A population-based study in a Swedish region. *Journal of Affective Disorders* 2021;287:165-73.
27. Cox JL, Holden JM, Sagovsky R. Detection of postnatal depression. Development of the 10-item Edinburgh Postnatal Depression Scale. *Br J Psychiatry* 1987;150:782-6. doi: 10.1192/bjp.150.6.782 [published Online First: 1987/06/01]
28. Socialstyrelsen. Vägledning för barnhälsovården, 2014.
29. Massoudi P, Wickberg B, Hwang P. Screening for postnatal depression in Swedish child health care. *Acta Paediatr* 2007;96(6):897-901. doi: 10.1111/j.1651-2227.2007.00292.x [published Online First: 2007/06/01]
30. Yonkers KA, Smith MV, Gotman N, et al. Typical somatic symptoms of pregnancy and their impact on a diagnosis of major depressive disorder. *General hospital psychiatry* 2009;31(4):327-33. doi: 10.1016/j.genhosppsych.2009.03.005
31. Byatt N, Biebel K, Lundquist RS, et al. Patient, provider, and system-level barriers and facilitators to addressing perinatal depression. *Journal of Reproductive and Infant Psychology* 2012;30(5):436-49. doi: https://doi.org/10.1080/02646838.2012.743000

For peer review only - http://bmjopen.bmj.com/site/about/guidelines.xhtml

For peer review only

32. Shiffman S, Stone AA, Hufford MR. Ecological momentary assessment. *Annu Rev Clin Psychol* 2008;4:1-32. doi: 10.1146/annurev.clinpsy.3.022806.091415 [published Online First: 2008/05/30]

33. Halfin A. Depression: the benefits of early and appropriate treatment. American Journal of Managed Care. 2007/12/06 ed, 2007:S92-7.

34. Jiménez-Serrano S, Tortajada S, García-Gómez JM. A mobile health application to predict postpartum depression based on machine learning. *Telemedicine and e-Health* 2015;21(7):567-74. doi: 10.1089/tmj.2014.0113

35. Dennis CLE, Janssen PA, Singer J. Identifying women at-risk for postpartum depression in the immediate postpartum period. *Acta Psychiatrica Scandinavica* 2004;110(5):338-46. doi: 10.1111/J.1600-0447.2004.00337.X

36. Ruyak SL, Lowe NK, Corwin EJ, et al. Prepregnancy Obesity and a Biobehavioral Predictive Model for Postpartum Depression. *Journal of Obstetric, Gynecologic & Neonatal Nursing* 2016;45(3):326-38. doi: 10.1016/J.JOGN.2015.12.013

37. Zhang W, Liu H, Silenzio VMB, et al. Machine Learning Models for the Prediction of Postpartum Depression: Application and Comparison Based on a Cohort Study. *JMIR Med Inform* 2020;8(4):e15516. doi: 10.2196/15516 [published Online First: 2020/05/01]

38. Cooper PJ, Murray L, Hooper R, et al. The development and validation of a predictive index for postpartum depression. *Psychological Medicine* 1996;26(3):627-34. doi: 10.1017/S0033291700035698

39. Andersson S, Bathula DR, Iliadis SI, et al. Predicting women with depressive symptoms postpartum with machine learning methods. *Scientific Reports 2021 11:1* 2021;11(1):1-15. doi: 10.1038/s41598-021-86368-y

40. De Choudhury M, Counts S, Horvitz E. Predicting postpartum changes in emotion and behavior via social media. *Conference on Human Factors in Computing Systems - Proceedings* 2013:3267-76. doi: 10.1145/2470654.2466447

41. De Choudhury M, Counts S, Horvitz EJ, et al. Characterizing and Predicting Postpartum Depression from Shared Facebook Data. *Proceedings of the 17th ACM conference on Computer supported cooperative work & social computing* 2014 doi: 10.1145/2531602

For peer review only

42. Fatima I, Abbasi BUD, Khan S, et al. Prediction of postpartum depression using machine learning techniques from social media text. *Expert Systems* 2019;36(4):e12409-e09. doi: https://doi.org/10.1111/exsy.12409
43. Mehta D, Newport DJ, Frishman G, et al. Early predictive biomarkers for postpartum depression point to a role for estrogen receptor signaling. *Psychol Med* 2014;44(11):2309-22. doi: 10.1017/S0033291713003231 [published Online First: 2014/02/06]
44. Blencowe H, Cousens S, Chou D, et al. Born too soon: the global epidemiology of 15 million preterm births. *Reprod Health* 2013;10 Suppl 1:S2. doi: 10.1186/1742-4755-10-S1-S2 [published Online First: 2014/03/15]
45. Serenius F, Källén K, Blennow M, et al. Neurodevelopmental outcome in extremely preterm infants at 2.5 years after active perinatal care in Sweden. *Jama* 2013;309(17):1810-20. doi: 10.1001/jama.2013.3786
46. Jacobsson B, Pettersson K, Modzelewska D, et al. Förtidsbörd största perinatala problemet. Läkartidningen, 2019.
47. Räisänen S, Gissler M, Saari J, et al. Contribution of risk factors to extremely, very and moderately preterm births–register-based analysis of 1,390,742 singleton births. *PLoS One* 2013;8(4):e60660. doi: https://doi.org/10.1371/journal.pone.0060660
48. Staneva A, Bogossian F, Pritchard M, et al. The effects of maternal depression, anxiety, and perceived stress during pregnancy on preterm birth: A systematic review. *Women and Birth* 2015;28(3):179-93. doi: 10.1016/J.WOMBI.2015.02.003
49. Goldenberg RL, Culhane JF, Iams JD, et al. Epidemiology and causes of preterm birth. *The lancet* 2008;371(9606):75-84. doi: https://doi.org/10.1016/S0140-6736(08)60074-4
50. Fransson E, Ortenstrand A, Hjelmstedt A. Antenatal depressive symptoms and preterm birth: a prospective study of a Swedish national sample. *Birth* 2011;38(1):10-6. doi: 10.1111/j.1523-536X.2010.00441.x [published Online First: 2011/02/22]
51. Eklundh A, Grunewald C. Handläggning vid hotande förtidsbörd. Läkartidningen. klinik och vetenskap ed, 2011.

For peer review only - http://bmjopen.bmj.com/site/about/guidelines.xhtml

52. Health NIo. What are the risk factors for preterm labor and birth? 2019 [Available from: https://www.nichd.nih.gov/health/topics/preterm/conditioninfo/who_risk accessed October, 2021.

53. Christian LM. Psychoneuroimmunology in pregnancy: immune pathways linking stress with maternal health, adverse birth outcomes, and fetal development. *Neurosci Biobehav Rev* 2012;36(1):350-61. doi: 10.1016/j.neubiorev.2011.07.005 [published Online First: 2011/07/27]

54. Medley N, Vogel JP, Care A, et al. Interventions during pregnancy to prevent preterm birth: an overview of Cochrane systematic reviews. *Cochrane Database of Systematic Reviews* 2018(11) doi: https://doi.org/10.1002/14651858.CD012505.pub2

55. Ferrero DM, Larson J, Jacobsson B, et al. Cross-country individual participant analysis of 4.1 million singleton births in 5 countries with very high human development index confirms known associations but provides no biologic explanation for 2/3 of all preterm births. *PloS one* 2016;11(9):e0162506. doi: https://doi.org/10.1371/journal.pone.0162506

56. Jacobsson B. Prediktion, prevention och behandlingsmetoder. Läkartidningen, 2019.

57. Varli IH, Wollmann CL, Sandström A. Hotande förtidsbörd/prematurbörd/för tidig födsel: Internetmedicin.se; 2019 [Available from: https://www.internetmedicin.se/behandlingsoversikter/gynekologi-obstetrik/prematura-sammandragningar/.

58. Torous J, Kiang MV, Lorme J, et al. New Tools for New Research in Psychiatry: A Scalable and Customizable Platform to Empower Data Driven Smartphone Research. *JMIR Mental Health* 2016;3(2):e16. doi: 10.2196/mental.5165

59. Meyerowitz-Katz G, Ravi S, Arnolda L, et al. Rates of Attrition and Dropout in App-Based Interventions for Chronic Disease: Systematic Review and Meta-Analysis. *Journal of Medical Internet Research* 2020;22(9):e20283. doi: 10.2196/20283 [published Online First: 2020/09/30]

60. Press G. 12 Big Data Definitions: What's Yours? Forbes, 2014.

61. Pastorino R, De Vito C, Migliara G, et al. Benefits and challenges of Big Data in healthcare: An overview of the European initiatives. *European Journal of Public Health* 2019;29(Supplement_3):23-27. doi: 10.1093/eurpub/ckz168

For peer review only - http://bmjopen.bmj.com/site/about/guidelines.xhtml

62. Torous J, Staples P, Onnela JP. Realizing the potential of mobile mental health: new methods for new data in psychiatry. *Curr Psychiatry Rep* 2015;17(8):602. doi: 10.1007/s11920-015-0602-0 [published Online First: 2015/06/16]
63. Olsen J, Melbye M, Olsen SF, et al. The Danish National Birth Cohort: its background, structure and aim. *Scand J Public Health* 2001;29(4):300-7. doi: 10.1177/14034948010290040201 [published Online First: 2002/01/05]
64. Magnus P, Birke C, Vejrup K, et al. Cohort Profile Update: The Norwegian Mother and Child Cohort Study (MoBa). *Int J Epidemiol* 2016;45(2):382-8. doi: 10.1093/ije/dyw029 [published Online First: 2016/04/12]
65. Stoltenberg C, Schjolberg S, Bresnahan M, et al. The Autism Birth Cohort: a paradigm for gene-environment-timing research. *Mol Psychiatry* 2010;15(7):676-80. doi: 10.1038/mp.2009.143 [published Online First: 2010/06/24]
66. Axfors C, Bränn E, Henriksson HE, et al. Cohort profile: The Biology, Affect, Stress, Imaging and Cognition (BASIC) study on perinatal depression in a population-based Swedish cohort. *BMJ Open* 2019;9(10):1-11. doi: 10.1136/bmjopen-2019-031514
67. Onnela JP, Rauch SL. Harnessing Smartphone-Based Digital Phenotyping to Enhance Behavioral and Mental Health: Nature Publishing Group, 2016:1691-96.
68. Shouval R, Bondi O, Mishan H, et al. Application of machine learning algorithms for clinical predictive modeling: a data-mining approach in SCT. *Bone Marrow Transplant* 2014;49(3):332-7. doi: 10.1038/bmt.2013.146 [published Online First: 2013/10/08]
69. Barnett I, Torous J, Staples P, et al. Relapse prediction in schizophrenia through digital phenotyping: a pilot study. *Neuropsychopharmacology* 2018;43(8):1660-66. doi: 10.1038/s41386-018-0030-z [published Online First: 2018/03/08]
70. Zulueta J, Piscitello A, Rasic M, et al. Predicting Mood Disturbance Severity with Mobile Phone Keystroke Metadata: A BiAffect Digital Phenotyping Study. *J Med Internet Res* 2018;20(7):e241. doi: 10.2196/jmir.9775 [published Online First: 2018/07/22]

For peer review only

For peer review only - http://bmjopen.bmj.com/site/about/guidelines.xhtml

71. Hussain-Shamsy N, Shah A, Vigod SN, et al. Mobile Health for Perinatal Depression and Anxiety: Scoping Review. *J Med Internet Res* 2020;22(4):e17011-e11. doi: 10.2196/17011

72. Hahn L, Eickhoff SB, Habel U, et al. Early identification of postpartum depression using demographic, clinical, and digital phenotyping. *Translational psychiatry* 2021;11(1):1-10. doi: https://doi.org/10.1038/s41398-021-01245-6

73. Graham S, Depp C, Lee EE, et al. Artificial Intelligence for Mental Health and Mental Illnesses: an Overview: Current Medicine Group LLC 1, 2019:116-16.

74. Micheli-Tzanakou E. Artificial neural networks: an overview. *Network: Computation in Neural Systems* 2011;22(1-4):208-30. doi: https://doi.org/10.3109/0954898X.2011.638355

75. LeCun Y, Bengio Y, Hinton G. Deep learning. *Nature* 2015;521(7553):436-44. doi: 10.1038/nature14539 [published Online First: 2015/05/29]

76. Miotto R, Wang F, Wang S, et al. Deep learning for healthcare: Review, opportunities and challenges. *Briefings in Bioinformatics* 2018;19(6):1236-46. doi: 10.1093/bib/bbx044

77. Topol EJ. High-performance medicine: the convergence of human and artificial intelligence. *Nat Med* 2019;25(1):44-56. doi: 10.1038/s41591-018-0300-7 [published Online First: 2019/01/09]

78. Yahata N, Kasai K, Kawato M. Computational neuroscience approach to biomarkers and treatments for mental disorders. *Psychiatry Clin Neurosci* 2017;71(4):215-37. doi: 10.1111/pcn.12502 [published Online First: 2016/12/30]

79. Joshi J, Goecke R, Alghowinem S, et al. Multimodal assistive technologies for depression diagnosis and monitoring. *Journal on Multimodal User Interfaces* 2013;7(3):217-28.

80. SBU. Diagnostik och uppföljning av förstämningssyndrom: en systematisk litteraturöversikt. In: Ekselius L, ed.: Statens beredning för medicinskt utvärdering (SBU), 2012.

81. Levis B, Negeri Z, Sun Y, et al. Accuracy of the Edinburgh Postnatal Depression Scale (EPDS) for screening to detect major depression among pregnant and postpartum women: systematic review and meta-analysis of individual participant data. *bmj* 2020;371 doi: https://doi.org/10.1136/bmj.m4022

For peer review only - http://bmjopen.bmj.com/site/about/guidelines.xhtml

82. France DJ, Shiavi RG, Silverman S, et al. Acoustical properties of speech as indicators of depression and suicidal risk. *IEEE transactions on Biomedical Engineering* 2000;47(7):829-37. doi: 10.1109/10.846676

83. Yang Y, Fairbairn C, Cohn JF. Detecting Depression Severity from Vocal Prosody. *IEEE Trans Affect Comput* 2013;4(2):142-50. doi: 10.1109/T-AFFC.2012.38 [published Online First: 2013/04/01]

84. An investigation of depressed speech detection: Features and normalization. Twelfth Annual Conference of the International Speech Communication Association; 2011.

85. On the relative importance of vocal source, system, and prosody in human depression. 2013 IEEE International Conference on Body Sensor Networks; 2013. IEEE.

86. Saeb S, Zhang M, Karr CJ, et al. Mobile Phone Sensor Correlates of Depressive Symptom Severity in Daily-Life Behavior: An Exploratory Study. *J Med Internet Res* 2015;17(7):e175. doi: 10.2196/jmir.4273 [published Online First: 2015/07/17]

87. Trajectories of depression: unobtrusive monitoring of depressive states by means of smartphone mobility traces analysis. Proceedings of the 2015 ACM international joint conference on pervasive and ubiquitous computing; 2015.

88. Cotten SR, Ford G, Ford S, et al. Internet use and depression among older adults. *Computers in human behavior* 2012;28(2):496-99. doi: https://doi.org/10.1016/j.chb.2011.10.021

89. Katikalapudi R, Chellappan S, Montgomery F, et al. Associating internet usage with depressive behavior among college students. *IEEE Technology and Society Magazine* 2012;31(4):73-80. doi: 10.1109/MTS.2012.2225462

90. Rozgonjuk D, Levine JC, Hall BJ, et al. The association between problematic smartphone use, depression and anxiety symptom severity, and objectively measured smartphone use over one week. *Computers in Human Behavior* 2018;87:10-17.

91. Elhai JD, Tiamiyu MF, Weeks JW, et al. Depression and emotion regulation predict objective smartphone use measured over one week. *Personality and Individual Differences* 2018;133:21-28. doi: https://doi.org/10.1016/j.paid.2017.04.051

For peer review only

92. Aledavood T, Torous J, Triana Hoyos AM, et al. Smartphone-Based Tracking of Sleep in Depression, Anxiety, and Psychotic Disorders. *Curr Psychiatry Rep* 2019;21(7):49. doi: 10.1007/s11920-019-1043-y [published Online First: 2019/06/05]

93. DeMasi O, Feygin S, Dembo A, et al. Well-Being Tracking via Smartphone-Measured Activity and Sleep: Cohort Study. *JMIR Mhealth Uhealth* 2017;5(10):e137. doi: 10.2196/mhealth.7820 [published Online First: 2017/10/07]

94. Torous J, Staples P, Barnett I, et al. Characterizing the clinical relevance of digital phenotyping data quality with applications to a cohort with schizophrenia. *NPJ Digit Med* 2018;1:15. doi: 10.1038/s41746-018-0022-8 [published Online First: 2018/04/06]

95. Multimodal measurement of depression using deep learning models. Proceedings of the 7th Annual Workshop on Audio/Visual Emotion Challenge; 2017.

96. Towards multi-modal anticipatory monitoring of depressive states through the analysis of human-smartphone interaction. Proceedings of the 2016 ACM international joint conference on pervasive and ubiquitous computing: adjunct; 2016.

97. Mental health analysis using deep learning for feature extraction. Proceedings of the ACM India Joint International Conference on Data Science and Management of Data; 2018.

98. Mehrotra A, Musolesi M. Using autoencoders to automatically extract mobility features for predicting depressive states. *Proceedings of the ACM on Interactive, Mobile, Wearable and Ubiquitous Technologies* 2018;2(3):1-20. doi: https://doi.org/10.1145/3264937

99. Atrey PK, Hossain MA, El Saddik A, et al. Multimodal fusion for multimedia analysis: a survey. *Multimedia systems* 2010;16(6):345-79. doi: 10.1007/s00530-010-0182-0

100. Fransson E, Karalexi M, Kimmel M, et al. Mental health among pregnant women during the pandemic in Sweden, a mixed methods approach using data from the Mom2B mobile application for research. *MedRxiv* 2020 doi: https://doi.org/10.1101/2020.12.18.20248466

For peer review only - http://bmjopen.bmj.com/site/about/guidelines.xhtml

101. Zhou C, Hu H, Wang C, et al. The effectiveness of mHealth interventions on postpartum depression: A systematic review and meta-analysis. *J Telemed Telecare* 2020:1357633X20917816. doi: 10.1177/1357633X20917816 [published Online First: 2020/04/21]

102. Bruno A, Celebre L, Mento C, et al. When fathers begin to falter: a comprehensive review on paternal perinatal depression. *International journal of environmental research and public health* 2020;17(4):1139. doi: https://doi.org/10.3390/ijerph17041139

103. Topp CW, Ostergaard SD, Sondergaard S, et al. The WHO-5 Well-Being Index: a systematic review of the literature. *Psychother Psychosom* 2015;84(3):167-76. doi: 10.1159/000376585 [published Online First: 2015/04/04]

104. Zigmond AS, Snaith RP. The hospital anxiety and depression scale. *Acta Psychiatr Scand* 1983;67(6):361-70. doi: 10.1111/j.1600-0447.1983.tb09716.x [published Online First: 1983/06/01]

105. Craig CL, Marshall AL, Sjostrom M, et al. International physical activity questionnaire: 12-country reliability and validity. *Med Sci Sports Exerc* 2003;35(8):1381-95. doi: 10.1249/01.MSS.0000078924.61453.FB

106. Rosen R, Brown C, Heiman J, et al. The Female Sexual Function Index (FSFI): a multidimensional self-report instrument for the assessment of female sexual function. *J Sex Marital Ther* 2000;26(2):191-208. doi: 10.1080/009262300278597 [published Online First: 2000/04/27]

107. Eklund M, Backstrom M, Tuvesson H. Psychometric properties and factor structure of the Swedish version of the Perceived Stress Scale. *Nord J Psychiatry* 2014;68(7):494-9. doi: 10.3109/08039488.2013.877072 [published Online First: 2014/01/28]

108. Rosengren A, Orth-Gomer K, Wedel H, et al. Stressful life events, social support, and mortality in men born in 1933. *BMJ* 1993;307(6912):1102-5. doi: 10.1136/bmj.307.6912.1102 [published Online First: 1993/10/30]

109. Greenwald R, Rubin A. Assessment of posttraumatic symptoms in children: Development and preliminary validation of parent and child scales. *Research on Social Work Practice* 1999;9(1):61-75. doi: https://doi.org/10.1177/104973159900900105

For peer review only - http://bmjopen.bmj.com/site/about/guidelines.xhtml

110. Hildingsson I, Rubertsson C, Karlstrom A, et al. Exploring the Fear of Birth Scale in a mixed population of women of childbearing age-A Swedish pilot study. *Women Birth* 2018;31(5):407-13. doi: 10.1016/j.wombi.2017.12.005

111. Wagnild GM, Young HM. Development and psychometric evaluation of the Resilience Scale. *J Nurs Meas* 1993;1(2):165-78. [published Online First: 1993/01/01]

112. Eriksson M, Lindstrom B. Validity of Antonovsky's sense of coherence scale: a systematic review. *J Epidemiol Community Health* 2005;59(6):460-6. doi: 10.1136/jech.2003.018085

113. Dennis C, Boyce P. Further psychometric testing of a brief personality scale to measure vulnerability to postpartum depression. *Journal of Psychosomatic Obstetrics & Gynecology* 2004;25(3-4):305-11. doi: https://doi.org/10.1080/01674820400017962

114. Brennan KA, Clark CL, Shaver P. Self-report measures of adult romantic attachment. *Attachment theory and close relationships* 1998:46-76.

115. Morelius E, Elander A, Saghamre E. A Swedish translation and validation of the Mother-to-Infant Bonding Scale. *Scand J Public Health* 2021;49(4):465-70. doi: 10.1177/1403494820910336 [published Online First: 2020/03/12]

116. Brockington IF, Fraser C, Wilson D. The Postpartum Bonding Questionnaire: a validation. *Arch Womens Ment Health* 2006;9(5):233-42. doi: 10.1007/s00737-006-0132-1

117. Putnam SP, Helbig AL, Gartstein MA, et al. Development and assessment of short and very short forms of the Infant Behavior Questionnaire–Revised. *Journal of personality assessment* 2014;96(4):445-58.

118. Bates JE, Freeland CA, Lounsbury ML. Measurement of infant difficultness. *Child Dev* 1979;50(3):794-803. doi: https://doi.org/10.2307/1128946

119. Burman M, Norlander A-K, Carlbring P, et al. Närmare varandra: Nio veckor till en starkare parrelation: Natur & Kultur 2018.

LEGENDS FOR FIGURES AND ILLUSTRATIONS

Figure 1. *From top to bottom, the gray content blocks in the main column represent installed apps (downloads of the Mom2B app by unique users from either app store (iOS) or google play (Android), registered users (individuals that have submitted registration information in the app), signed consents (registered users that have consented to contributing data, and signed these consents electronically), and finally, data (participants with signed consents*

For peer review only - http://bmjopen.bmj.com/site/about/guidelines.xhtml

that, at minimum, have at least once completed the EPDS[27]. The latter two blocks also illustrate the signed consents and available data, respectively, by type of data (survey, voice and passive data). The intersections of the Venn diagrams are non-exclusive, meaning that the number count in the intersection of surveys and passive data, for example, can include individuals who have also contributed voice-recordings. This flowchart reflects data last downloaded on September 6, 2021.

Figure 2. *Flow of data from user to servers for storage and analysis. Data passes through secure servers accessible only by authorized members of the Mom2B team, and can be decrypted for analysis in Bianca when needed.*

Figure 3. *A multimodal ML model for PND diagnosis. The extracted features can be classified into three categories: acoustic signals, time-series features and categorical features. We can then determine the most suitable model for each category. For example, for acoustic signals, we would apply Convolutional Neural Network; for time-series data, we would apply recurrent neural network such as long short memory; and for numerical variables, we would apply DNNs such as transformers, or traditional models like extremely randomized trees, gradient boost trees, etc. These models can yield high-dimension representations of multimodal features. After feature fusion, the integrated features will be fed in to another neural network for prediction.*

For peer review only

For peer review only - http://bmjopen.bmj.com/site/about/guidelines.xhtml

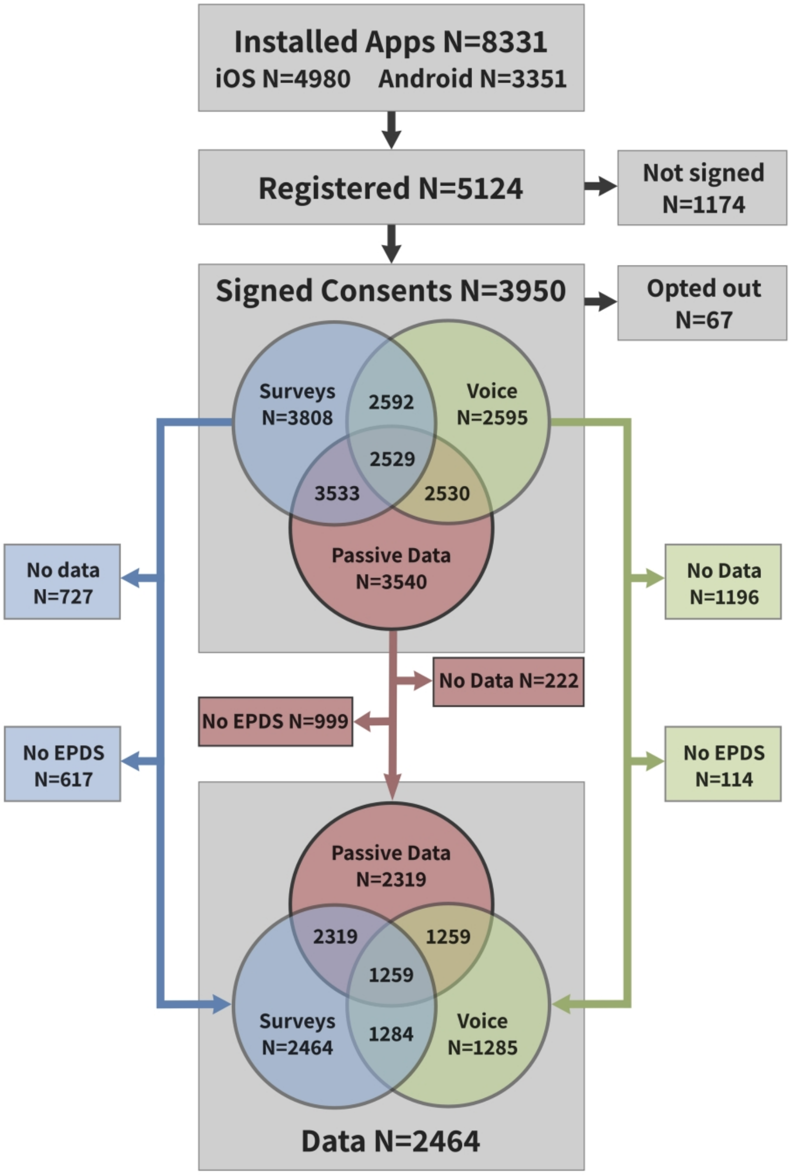

From top to bottom, the gray content blocks in the main column represent installed apps (downloads of the Mom2B app by unique users from either app store (iOS) or google play (Android), registered users (individuals that have submitted registration information in the app), signed consents (registered users that have consented to contributing data, and signed these consents electronically), and finally, data (participants with signed consents that, at minimum, have at least once completed the EPDS[27]. The latter two blocks also illustrate the signed consents and available data, respectively, by type of data (survey, voice and passive data). The intersections of the Venn diagrams are non-exclusive, meaning that the number count in the intersection of surveys and passive data, for example, can include individuals who have also contributed voice-recordings. This flowchart reflects data last downloaded on September 6, 2021.

105x144mm (600 x 600 DPI)

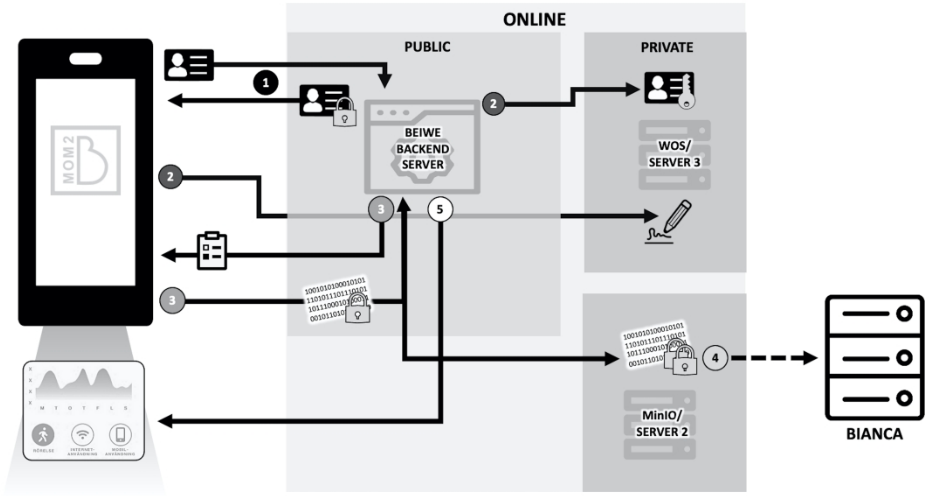

Flow of data from user to servers for storage and analysis. Data passes through secure servers accessible only by authorized members of the Mom2B team, and can be decrypted for analysis in Bianca when needed.

639x319mm (300 x 300 DPI)

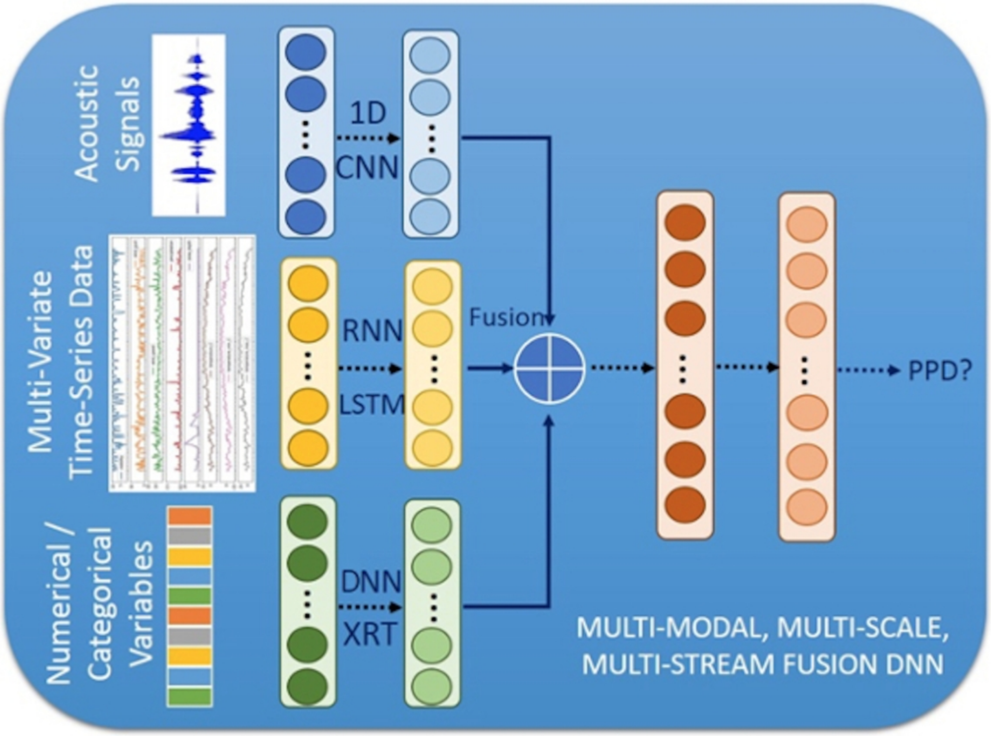

A multimodal ML model for PND diagnosis. The extracted features can be classified into three categories: acoustic signals, time-series features and categorical features. We can then determine the most suitable model for each category. For example, for acoustic signals, we would apply Convolutional Neural Network; for time-series data, we would apply recurrent neural network such as long short memory; and for numerical variables, we would apply DNNs such as transformers, or traditional models like extremely randomized trees, gradient boost trees, etc. These models can yield high-dimension representations of multimodal features. After feature fusion, the integrated features will be fed in to another neural network for prediction.

210x155mm (300 x 300 DPI)

BMJ Open

Predicting perinatal health outcomes using smartphone-based digital phenotyping and machine learning in a prospective Swedish cohort (Mom2B): study protocol

Journal:	*BMJ Open*
Manuscript ID	bmjopen-2021-059033.R1
Article Type:	Protocol
Date Submitted by the Author:	21-Mar-2022
Complete List of Authors:	Bilal, Ayesha; Uppsala University, Department of Medical Sciences; Uppsala Universitet, Centre for Women's Mental Health during the Reproductive Lifespan (Womher)<br>Fransson, Emma; Uppsala University, Department of Women’s and Children’s health; Karolinska Institute, Centre for Translational Microbiome Research, Department of Microbiology, Tumor and Cell Biology<br>Bränn, Emma; Uppsala University, Department of Women's and Children's Health<br>Eriksson, Allison; Uppsala University, Department of Women’s and Children’s health; Uppsala University, Centre for Women's Mental Health during the Reproductive Lifespan (Womher)<br>Zhong, Mengyu; Uppsala University, Department of Information Technology; Uppsala Universitet, Centre for Women's Mental Health during the Reproductive Lifespan (Womher)<br>Gidén, Karin; Uppsala University, Department of Women’s and Children’s health<br>Elofsson, Ulf; Uppsala University, Department of Medical Sciences, Psychiatry; Uppsala University, Department of Women's and Children's health<br>Axfors, Cathrine; Uppsala Universitet, Department of Women’s and Children’s Health,<br>Skalkidou, Alkistis; Uppsala Universitet, Department of Women's and Children's health<br>Papadopoulos, Fotios C.; Uppsala Universitet, Department of Medical Sciences
**Primary Subject Heading**:	Mental health
Secondary Subject Heading:	Obstetrics and gynaecology, Mental health
Keywords:	Depression & mood disorders < PSYCHIATRY, MENTAL HEALTH, Maternal medicine < OBSTETRICS, PERINATOLOGY, PREVENTIVE MEDICINE, Anxiety disorders < PSYCHIATRY

For peer review only - http://bmjopen.bmj.com/site/about/guidelines.xhtml

Predicting perinatal health outcomes using smartphone-based digital phenotyping and machine learning in a prospective Swedish cohort (Mom2B): study protocol

Ayesha M Bilal[1, 2], Emma Fransson[3, 4], Emma Bränn[3], Allison Eriksson[2, 3], Mengyu Zhong[2, 5], Karin Gidén[3], Ulf Elofsson[1, 3], Cathrine Axfors[3], Alkistis Skalkidou[3*], Fotios C Papadopoulos[1*]

1. Department of Medical Sciences, Psychiatry, Uppsala University, Uppsala, Sweden
2. Centre for Women's Mental Health during the Reproductive Lifespan (Womher), Uppsala University, Uppsala, Sweden.
3. Department of Women's and Children's health, Uppsala University, Uppsala, Sweden
4. Centre for Translational Microbiome Research, Department of Microbiology, Tumor and Cell Biology, Karolinska Institutet, Stockholm, Sweden
5. Department of Information Technology, Uppsala University, Uppsala, Sweden

*equal contribution

Correspondence: Ayesha Mae Bilal
Akademiska sjukhuset 751 85 Uppsala
Email: ayesha.bilal@neuro.uu.se

Word count: 4,280

For peer review only - http://bmjopen.bmj.com/site/about/guidelines.xhtml

ABSTRACT

Introduction: Perinatal complications, such as perinatal depression and preterm birth, are major causes of morbidity and mortality for the mother and the child. Prediction of high risk can allow for early delivery of existing interventions for prevention. This ongoing study aims to use digital phenotyping data from the Mom2B smartphone application to develop models to predict women at high risk for mental and somatic complications.

Methods and analysis: All Swedish-speaking women over 18 years, who are either pregnant or within three months postpartum are eligible to participate by downloading the Mom2B smartphone app. We aim to recruit at least 5000 participants with completed outcome measures. Throughout the pregnancy and within the first year postpartum, both active and passive data are collected via the app in an effort to establish a participant's digital phenotype. Active data collection consists of surveys related to participant background information, mental and physical health, lifestyle, and social circumstances, as well as voice recordings. Participants' general smartphone activity, geographical movement patterns, social media activity, and cognitive patterns can be estimated through passive data collection from smartphone sensors and activity logs. The outcomes will be measured using surveys, such as the Edinburgh Postnatal Depression Scale (EPDS), and through linkage to national registers, from where information on registered clinical diagnoses and received care, including prescribed medication, can be obtained. Advanced machine learning and deep learning techniques will be applied to these multimodal data in order to develop accurate algorithms for the prediction of perinatal depression and preterm birth. In this way, earlier intervention may be possible.

Ethics and dissemination: Ethical approval has been obtained from the Swedish Ethical Review Authority (dnr: 2019/01170, with amendments), and the project fully fulfills the GDPR requirements. All participants provide consent to participate and can withdraw their participation at any time. Results from this project will be disseminated in international peer-reviewed journals and presented in relevant conferences.

Keywords: perinatal depression, preterm birth, prediction, digital phenotyping, smartphone application, machine learning, deep learning

STRENGTHS AND LIMITATIONS OF THIS STUDY

* The study collects large-scale, temporally sensitive data regarding the user's behaviors in the real world.
* End-users' feedback collected allows for app updates and improvements.
* The passive data collection is expected to have lower attrition rate.
* The active data collection is prone to suffer from a higher attrition rate.
* There are high costs associated with recruiting participants and maintaining frontend and backend for the smartphone app.

For peer review only - http://bmjopen.bmj.com/site/about/guidelines.xhtml

INTRODUCTION

Optimal maternal health is important throughout pregnancy, childbirth and the postnatal period, to ensure the full potential for the mother, infant and family to get a good start[1]. Two health conditions that are important to address in order to reach a goal of good maternal health is perinatal depression (PND) and preterm birth (PTB), both affecting about every tenth pregnancy worldwide[2, 3].

Perinatal depression

PND is an episode of major depression with onset anytime during pregnancy and up to four weeks postpartum[4], although in research settings, a period of up to one year postpartum is often considered[5]. Antenatal depression (AND) affects between 7-13% of pregnant women[6], and postpartum depression (PPD) is estimated to affect between 10-20% of all newly-delivered mothers[2], while many women experience persistent depression throughout the perinatal period[7]. PND is distinct from “baby blues”, which are commonly experienced symptoms of low mood and anxiety that subside within two weeks postpartum. PND is both emotionally and physically debilitating like major depression, with additional risks related to the pregnancy and birth, such as PTB, low birth weight, preeclampsia and placental abnormalities[8-10]. Moreover, it is associated with retained maternal weight postpartum[11], decreased breastfeeding[12-14], poor maternal sleep[15], and poor perinatal quality of life[16]. PND can also compromise the critical mother-infant bond, as it affects the mother’s caregiving abilities and adaptation to the maternal role[16, 17], and has a long-term impact on the child’s cognitive, emotional and behavioral development[18, 19]. Furthermore, PND can be characterized by the occurrence of self-harm thoughts, which are linked to long-term somatic and psychiatric morbidity[20], and increased maternal mortality from suicide in the first year postpartum[21].

The etiology of PND is multifactorial, including biological, genetic, psychological, and social factors, such as stressful life events, social support, domestic violence, childhood adversity, history of depression and anxiety, low self-esteem, and even personality traits like resilience[15, 22-24]. Despite this knowledge, detecting PND remains a challenge for the healthcare system, with one review finding that around 30-70% of cases go undetected and only 15% receive adequate treatment[25, 26].

Current screening protocols include the Edinburgh Postnatal Depression Scale (EPDS)[27] during postnatal visits to assess risk of PND[28]. However, early detection of PND has remained challenging for many reasons, including inconsistencies in screening practices[29], and failure to distinguish depressive symptoms due to their overlap with typical somatic experiences in the early postpartum period[30]. Furthermore, women may hesitate to seek care, possibly because of the depression itself, but also stigma and a fear of being judged as an imperfect mother, as well as concerns about antidepressant use during pregnancy and breastfeeding[31]. Besides, in-clinic screening frequently relies on retrospective self-reports of diagnostically relevant information, making it susceptible to errors and biases associated with autobiographical recollection[32].

Unquestionably, more efficient and effective methods for predicting PND in mothers are needed to enable early identification and intervention, thus improving prognosis, and reducing the burden of disease[33]. Previous studies that have attempted to develop predictive models of maternal depression primarily focus on the postpartum period only[34-39]. Few have used social media fingerprints[40-42] or biomarkers[36, 43] in their models, and these studies largely depended on psychometric self-reports and limited modalities. These

For peer review only - http://bmjopen.bmj.com/site/about/guidelines.xhtml

drawbacks compromise the predictive power of the models and illustrate why multivariate, real-time and unobtrusive approaches to data collection and symptom-monitoring must be encouraged to develop better predictive models.

Preterm birth

Among somatic pregnancy complications, PTB is a major cause of neonatal death, as well as of poor long-term health in children, affecting approximately 15 million babies worldwide each year[44, 45]. In Sweden, the preterm birth rate is about 6%[46], which is a relatively low number compared to the international average of over 10%[44]. Like PND, the etiology of PTB is multifactorial, including previous PTB, multifetal pregnancy, cervical insufficiency, intrauterine infections, vaginal bleeding in the second trimester, in vitro fertilization, primiparity, as well as maternal antenatal stress and depression[47-50]. In fact, many risk factors overlap between PND and PTB, such as childhood traumatic events or maltreatment, stressful life events, being single or lacking social support, being overweight, smoking, and low socioeconomic status[51, 52]. Inflammation has been suggested as a possible underlying pathway for both depression and preterm delivery[53].

There are evidence-based interventions for preventing or delaying PTB to optimize birth outcomes, such as smoking cessation, progesterone therapy, cerclage in women with cervical insufficiency, or antibiotics[51, 54]. However, a major obstacle for the success of these interventions is the etiological heterogeneity of PTB, which makes it extremely challenging to identify women at high risk. In fact, two-thirds of women who experience PTB do not present with any risk factors at all[55]. Available biological diagnostic tests for PTB (such as fetal fibronectin) lack sufficient positive prediction values[56]. Screening for cervical length is performed in Sweden for women with a history of previous PTB, however, this is not helpful in primiparous women[57].

It can be concluded that no single biomarker is sufficient for prediction; multimodal data, including psychosocial and behavioral factors, should, therefore, be the focus of prediction efforts.

Digital phenotyping and big data

Digital devices like smartphones allow us to capture moment-by-moment, objective data regarding the patient's experiences and functions in non-clinical settings. This process, known as *digital phenotyping*[58], allows us to collect two kinds of data: *active data* and *passive data*. Active data refers to data that requires user input, such as surveys and voice recordings. Passive data refers to automatically collected data from smartphone sensors and activity logs, which can be used to infer the user's mobility and sleep patterns, digital social activity, smartphone usage patterns, and even affective and cognitive changes.

The Mom2B smartphone app is developed using the Beiwe research platform (www.beiwe.org) from the Harvard School of Public Health. It can allow us to capture digital phenotyping data during the perinatal period with greater efficiency and temporal sensitivity as data collection occurs continuously and in real-world contexts, which minimizes the risk of recall biases. Such apps could also be integrated into the mother's perinatal care plan. One drawback of smartphone-based data collection, in general, is that attrition rate increases with longer follow-up times[59]; however, this can at least in part be compensated for by the continuous collection of passive data.

In fact, one of the biggest advantages of smartphone-based digital phenotyping is the ability to collect multivariate, high-volume data, known commonly as big data[60]. Big data is

For peer review only - http://bmjopen.bmj.com/site/about/guidelines.xhtml

excellent for healthcare research since it can facilitate a unique insight into risk factors and the development of better diagnostic frameworks[61]; however, the literature on big data approaches for psychiatric conditions, particularly perinatal mental health, is limited[62]. Nordic countries are in the forefront in this respect – with the Danish National Birth Cohort (DNBC)[63], the Norwegian MoBa[64] and ABC[65] studies and the Swedish BASIC study[66] - due to the availability of nationwide registers with comprehensive personal and medical information for all pregnant women in these countries. Nonetheless, register data, while valuable, lacks the multeity, continuity and veracity offered by digital phenotyping[67]. Furthermore, studies derived from these cohorts have largely relied on traditional statistical methods, which are limited in their ability to scale to large datasets and identify more subtle patterns in data[68].

To date, few studies have applied digital phenotyping for prediction of psychiatric conditions, such as relapse in schizophrenia[69] and severity of mood episodes in bipolar disorder[70]. In the context of PND, while smartphone apps are widely used, their application has been largely focused on screening and intervention[71]. Only two studies have applied digital phenotyping for predicting PPD[34, 72]. While these studies have reported encouraging results, their predictive ability is compromised due to limited modalities (using only active data in the form of questionnaires), infrequent measurement points, and usage of more traditional statistical methods. The Mom2B study combines nationwide health and pregnancy register data with active and passive data collected through smartphone-based digital phenotyping to objectively monitor indicators of PND in non-clinical contexts.

In order to harness the full potential of big data, more advanced analytical methods, such as machine learning (ML) and deep learning (DL), are ideal. ML is an artificial intelligence approach that refers to various methods of enabling an algorithm to identify and learn intricate patterns in data to predict outcomes[73]. Modern ML methods such as deep neural networks (DNNs) are uniquely suited to analyzing big datasets as they can detect complex, high-dimensional interactions and structured information, without guidance, that can then be used to train predictive algorithms. DNN models are comprised of multiple "hidden" processing layers, inspired by biological neural networks, consisting of a series of interconnected nodes that resemble neurons[74, 75].

Over the last decade, there has been a steady increase in the use of DL methods in medicine[76]. However, few studies have used ML for diagnosis or risk assessment in psychiatry, and those that do are often limited by modest sample sizes and modalities, or from using only traditional ML techniques[73, 77]. To our knowledge, Mom2B is the first study to adopt a big data approach and use multimodal digital phenotyping with advanced ML techniques to develop predictive algorithms for PND and preterm birth.

Objectives

Using large-scale, multimodal data collected through the Mom2B smartphone app, together with health and pregnancy information from national registers, the primary aim of this study is to assess the accuracy of advanced ML and DL methods in predicting development of PPD (1) in the third pregnancy trimester, using data from the first trimester, and (2) during the early and late postpartum period, using data collected throughout pregnancy and childbirth.

A secondary aim of this study is to apply advanced ML and DL techniques using the multimodal dataset to predict the risk of PTB.

METHODS AND ANALYSIS

For peer review only - http://bmjopen.bmj.com/site/about/guidelines.xhtml

Cohort description

Mom2B (www.mom2b.se) is a national ongoing smartphone app-based study; the app was launched at the end of November 2019 to App Store and Google Play. All Swedish-speaking women above the age of 18 owning a smartphone, who are either pregnant or within three months postpartum, are eligible to participate by registering and providing consent in the Mom2B app. Participating women are also asked for optional consent to be contacted for additional research studies within and from outside the Mom2B project (see Appendix A). Participant data is then linked to psychiatric and somatic health- and pregnancy-related information available from Swedish national registers.

We aim to recruit at least 5000 participants with completed outcome measures. Due to the complexity of ML methods, it is not possible to perform any traditional test of statistical power. However, based on previous studies[59, 78, 79] and conferring with experts in artificial intelligence, we estimated that this approximate number would give us enough material to build robust prediction models while accounting for attrition and the prevalence rates of the outcomes.

Information about the study is being disseminated on social media, and through posters and brochures sent to primary and maternal care centers across the country. Figure 1 illustrates Mom2B recruitment, data collection and opt-outs. Table 1 outlines participant characteristics of the existing Mom2B cohort based on users who have contributed relevant data, along with similar characteristics in the general Swedish population.

Characteristics	Mom2B (2020-2022) (n=3909)[a]			Sweden (2019)[b]	
	Available data (*n*)	Missing data (*n*)	n(%) or mean±sd	Available data (*n*)	n(%) or mean
**Maternal age (years)**	3430	479	31.2±4.4	113 816	30.7
**Country of origin**	3441	468		112 530	
Sweden			3177 (92.3)		78 033 (69.3)
Nordic countries except Sweden			40 (1.2)		1280 (1.1)
Europe except Nordic countries			116 (3.4)		9172 (8.2)
Outside Europe			108 (3.1)		24 045 (21.4)
**Education**	3444	465		107 711	
≤12 years			744 (21.6)		48 793 (45.3)
Post-secondary education			2700 (78.4)		58 918 (54.7)
**Employment before pregnancy**	1677	2232		113 147	
Working/student/parental leave			1626 (97)		103 967 (91.9)
Unemployed/sick leave			51 (3)		9180 (8.1)
**Smoking 3 months before pregnancy**	3041	868	441 (14.5)	110 991	11 765 (10.6)
**BMI before pregnancy (kg/m²)**	3353	556	25.5±5.3	108 929	
<18,5			70 (2.1)		2783 (2.5)
18,5 – 25			1815 (54.1)		59 384 (54.6)
25 - <30			923 (27.5)		29 636 (27.2)
≥30			545 (16.3)		17 126 (15.7)
**Primiparous**	3268	641	1188 (36.4)	113 816	48 473 (42.5)
**Cesarean section**	1356	639[c]	238 (17.5)	114 757	20 312 (17.7)
**Preterm delivery (<week 37)**	3311	598	190(5.7)	116 071	6502 (5.6)

Table 1. *Sociodemographic characteristics, pregnancy history and birth outcomes on participants in the Mom2B study and the general population of pregnant women in Sweden. Percentages are given in relation to available data from women.* [a] *Data downloaded on February 1, 2022.* [b] *Data retrieved from the Swedish Medical Birth Register and Swedish National Board of Health and Welfare from 2019.* [c] *Calculated using the confirmed number of women in the postpartum period only.*

Data collection

The Mom2B app collects three types of data: survey data, audio recordings, as well as passive data. Data can be collected from the first week of pregnancy, and up till week 52 after birth. Only data that participants have consented for are collected from the time they register to the study, and they can change their consent preferences anytime in the app if they wish to stop.

For peer review only - http://bmjopen.bmj.com/site/about/guidelines.xhtml

Surveys and questionnaires

The Mom2B app delivers a range of both validated and self-developed questionnaires 2-3 times per week on average, with a mean of 5 questions per survey. These questionnaires are used to collect information regarding the participant's mental and physical wellbeing, and history, personality, relationships, as well as perinatal and parenthood experiences. They include the EPDS, a ten-item self-report screening tool with good psychometric properties[80, 81], used as the primary outcome measure in this study to assess depressive symptoms throughout the study period. A summary of the timeline of validated and self-developed instruments, along with the number of occurrences of the survey throughout the study period, can be found in Figures 2 and 3 respectively.

Voice recordings

Voice acoustic qualities, such as pitch, speed, timing and timbre, have been used in previous research to successfully distinguish depressed from non-depressed individuals[82-85]. To collect voice data, the Mom2B app sends out a voice recording task asking the participant to record reading simple texts, numerical sequences or vocalizations every 2-4 weeks.

Passive data collection

Passive data that the user has provided consent for are continuously collected via the Mom2B app throughout the study period, and are used to infer the user's behavioral patterns. Some of these features are collected differently for iOS and Android users. The feature modalities are briefly explained below:

Mobility

Correlations have been demonstrated between a patient's geographical movement patterns and changes in depressive symptoms[86, 87]. Sixty seconds of GPS data are continuously collected every 10 minutes. Accelerator data are collected when the motion exceeds a certain threshold; motion activities, including being stationary, walking, running, cycling, and movement in a vehicle are recorded when the state changes. For iOS, we collect device motion to provide more detailed motion sensor data.

Data usage

Internet usage is the main feature of data usage. Various patterns of internet usage have been identified in relation to depression in different populations[88, 89], but not among women with PND. The Mom2B app records the accumulated upload and download rates together with timestamps. Another feature, reachability, records timestamped smartphone connectivity – whether the phone is connected to cellular network, Wi-Fi or neither. It also records a Wi-Fi log for Android phone, which includes anonymized MAC addresses' frequencies and signal strength (RSSI) of available wireless networks in the area.

Smartphone usage

General smartphone use has been found to correlate with sleep quality and depression[90, 91]. Phone power state, combined with mobility parameters, can reflect individual behavior like sleep patterns[92, 93]. To keep track of the use of the smartphone device, data are collected on screen activity, charging status, and device reboot.

For peer review only - http://bmjopen.bmj.com/site/about/guidelines.xhtml

Social media activity
Social media behavior has also been proven useful in detecting mental states. It has also been shown that reduced social activity on Facebook predicted symptoms of PPD[41]. Collected data consist of simple behavioral measures , such as posting, commenting or liking, together with their timestamps. Notably, we only measure activity levels, not information related to the text or image content of that activity, and participants are made aware of this when providing consent.

Survey metadata
App-based surveys make it possible to also collect metadata. This kind of behavioral metadata may contain clinically relevant information related to attention, processing speed and working memory capacity, and even any deterioration of psychiatric symptoms[58, 94]. We collect data on the time a survey was opened, time taken to answer each question and fully complete a survey, as well as any changes made in survey responses.

National Register data
Supplementary information will be accessed via the following Swedish national health and quality registers: the Medical Birth Register, the Pregnancy Register, the National Patient Register, the Prescribed Drug Register, and population censuses from Statistics Sweden. The accessed information includes records of perinatal complications such as PND, PTB, or any other complications considered important risk factors for the study outcomes, such as gestational diabetes, gestational hypertension, preeclampsia, prolonged delivery, severe lacerations, postpartum hemorrhage, induction of delivery, instrumental vaginal delivery, cesarean section, and small for gestational age.
Further, the mother's weight at enrollment in maternity care and at aftercare visits; calculated date of birth from last menstrual period and from ultrasound; and information on previous miscarriages, previous abortions, chronic diseases, fear of childbirth, involuntary infertility, gestational age at enrollment in maternity care, and fetal diagnostics will be obtained from the Medical Birth Register. Retrieved information also includes variables regarding the background, health, and lifestyle of the participant, for validation purposes of our self-report questionnaires, as well as psychiatric and somatic morbidity for up to 15 years after childbirth.

Data flow and storage
Figure 4 illustrates the data flow and storage process as follows:
1. Participants register to the study via the Mom2B app using their Swedish Social Security Number, which is encrypted in the device using a private key provided by the Beiwe backend server, and replaced by a random, pseudo-anonymized code number.
2. The decryption key, together with the participant consent information and electronic signatures, is stored in a private, write-only server at Uppsala University.
3. The app fetches surveys and voice recording tasks, and uploads data from participants to the backend server, where it is encrypted and sent to MinIO, a secure, cloud-based storage, where another layer of encryption is added. Passive data collected from the phone follow the same path.
4. From MinIO, all data are sent to Bianca, a private offline server, in both encrypted and decrypted forms for storage and analysis respectively.

For peer review only - http://bmjopen.bmj.com/site/about/guidelines.xhtml

5. The app provides weekly reports based on participant activity and fetches health-related information relating to the perinatal period the user is in, as well as Frequently Asked Questions about the study and perinatal health.

Preliminary data analysis strategy

The Mom2B dataset contains different modalities, including audio data, sensor data and survey data, which will be analyzed separately and then combined. We plan to utilize both traditional ML and DL techniques in order to determine reliable predictors of PPD and develop accurate predictive algorithms, and will report our findings following the best-fit current guidelines, such as the Transparent Reporting of a Multivariable Prediction Model for Individual Prognosis or Diagnosis (TRIPOD) statement[95].

Feature engineering

To handle this multimodal data, we will extract features for these modalities separately, using traditional feature engineering as well as DL techniques[96, 97]. An example of traditional feature engineering utilizes trajectories[87], which use mobility features, such as number of significant places visited, maximum distance, and standard deviation. However, DL can be used for extracting features in many other modalities, such as social media and audio data, which are not investigated widely in the area.

Feature selection and model selection

To analyze the multimodal Mom2B dataset, we will start with each modality separately. To reduce the possibility of potential overfitting, given the numerous features in our dataset, we will use Recursive Feature Elimination to obtain the optimal set of variables for further model development.

Previously, logistic regression, support vector machine (SVM), random forests, XGBoost and neural networks have been the most commonly used and efficient ML algorithms for prediction of PND[98]. An advantage of using such traditional machine learning methods is to give us a feature importance ranking, allowing us to identify stronger predictors. Using DL to analyze digital phenotyping data for evaluating risk of depression is a relatively novel approach compared to traditional ML models[73]. DL models have been shown to outperform traditional ML in various tasks involving complex datasets[99, 100], and can be combined with traditional ML in multimodal data mining tasks to further improve performance[97]. We will test and select the best performing ML models for each modality and determine strong predictors of PND.

Multimodal computational model

The multimodal data we collect are in different scales, dimensions and formats, which need to be harmonized before prediction[101]. Different models are better suited to perform on different modalities. To handle this complexity in multiple data modalities, we consider modality fusion during the development phase.

One example of a multimodal ML model is shown in Figure 5. The model is designed to detect potential depressive episodes based on multimodal data collected in the Mom2B app. Pre-processed data in three modalities are fed into models and intermediate representations are then fused together and fed in as input features of a classification model.

Evaluation metrics

For peer review only - http://bmjopen.bmj.com/site/about/guidelines.xhtml

Our data will be split into a training dataset, for analysis, and a test dataset, to assess model performance. We consider using multiple evaluation metrics, including Area Under the ROC Curve, specificity, sensitivity, positive predictive value, negative predictive value, balanced accuracy, and F1 score, as these measurements vary in importance according to the setting and goal of the final algorithm. Thereby, we can compare the performance of traditional ML with DL, and the different assemblies of models, from different perspectives in the context of prediction of PND.

Patient and public involvement

A qualitative study is planned for exploring the attitudes and concerns of participating women towards the Mom2B app. Furthermore, an online survey will be sent to women who have had no recent activity on the app or withdrawn participation from the study. Direct contact with end-users and the ability to make changes to the app based on their feedback can enhance user experience and increase engagement. A representative from Mamma till Mamma, a non-profit organization in Sweden focused on perinatal mental wellbeing, serves on our advisory board. The organization has been involved in the piloting of our study and design of questionnaires, and currently supports us with recruitment. We plan to involve them in the dissemination of study results as well.

Sub-studies

In addition to predicting PND and PTB, the rich data collected from the Mom2B cohort will also be used to investigate further questions, mainly regarding the health of pregnant and postpartum women. Other planned areas of research are regarding the impact of early mother-infant separation and neonatal intensive care of the baby on the wellbeing of the mother; and sexual function and its potential correlates to depression and anxiety in the perinatal period.

Maternal depression and well-being during the Covid 19 pandemic

In the beginning of 2021, data collected from 1577 participants were used to assess depressive and anxiety symptoms, as well as wellbeing and life changes in pregnant women in Sweden during the COVID19 pandemic (from February 2020-March 2021)[102]. The Mom2B app enabled gathering psychiatric information at a national level during the pandemic, as well as passive data on mobility. Levels of perinatal affective symptoms and low wellbeing were elevated compared to previous years and to months with fewer cases. Similar apps can help healthcare providers and governmental bodies to monitor high-risk groups during crises in real time, as well as to adjust measures and the support offered.

ETHICS AND DISSEMINATION

Participants are informed about the aims of the study, and that the confidentiality and security of their data will be assured. All participants provide their consent to participate while registering to the study, and are informed that they can withdraw their participation at any time without giving a reason. Ethical approval has been obtained from the Swedish Ethical Review Authority (dnr: 2019/01170, with amendments) and the project fulfills GDPR requirements, including the processing, storage and protection of all data. Results will be continuously disseminated through international peer-reviewed journals, the project's website and social media channels, and presented in national and international conferences. All publications will be open access.

For peer review only - http://bmjopen.bmj.com/site/about/guidelines.xhtml

DISCUSSION

Strengths and limitations

Besides the utility of digital phenotyping in combination with the advanced analytical methods planned to be used, other strengths of the Mom2B study include the involvement of participants. Statistics based on WHO-5 and behavioral data (movement, internet usage, sleep etc.) collected from participants are sent to the user, allowing them to follow their wellbeing and activity as an incentive for continued participation. Weekly informational reports regarding common experiences and concerns for both the mother and child for that particular week of the perinatal period, based on information taken from 1177.se (Swedish healthcare service), are available to users and allow them to easily stay informed. As per standard guidelines[103], If participants receive a high score on the EPDS, they are prompted to contact their healthcare provider or emergency support services for support, and if unsure, they can contact the research team, which will help them find appropriate support for their needs. Continuous contact is maintained with participants until they find support.

The involvement of user organizations and an international advisory board further strengthen the study, by increasing the feasibility, the use of state-of-the-art methods and the potential for high acceptance by the end-users, which is especially important for future integration in regular clinical practice.

However, there are some limitations to acknowledge. Weekly reports and statistics are important in supporting and incentivizing users, but it is possible they may influence users' responses to certain questionnaires. To account for this, we consider including how often they are checked by users as a feature within our models. Furthermore, our app is available only in Swedish, which excludes a number of otherwise eligible participants, and the high costs for maintaining the technical infrastructure in the front- and back-end of the app, which require considerable funding. Attrition is also an issue, especially with data that requires active input from users. While we can attempt to combat this by improving the app based on user feedback, it is important to consider that attrition might also reflect the worsening of symptoms and be a predictor per se of clinical deterioration. It will be important to distinguish such participants and determine how to use attrition as a predictor variable.

Future perspectives

We are at the beginning of the smartphone-based research era, and future possibilities seem numerous. We intend to develop the Mom2B app in other languages, including English, to expand to a more diverse and wider population. If the app succeeds in developing good predictive models for PND, the research team anticipate that the app could be further developed to include evidence-based interventions[104]. Furthermore, since PND is much less understood in co-parents and improving the other parent's mental wellbeing is conducive to the health of the mother and the children as well[105], the app could be further developed to study co-parental PND. The Mom2B research team plans to further adapt the app to other research topics such as teenage and student mental health, and prediction of new episodes or self-harm in major depression.

CONTRIBUTORS

AS and FCP were involved in the conception and design of the study, revised the draft critically for intellectual content and approved the final manuscript. EB, EF and CA contributed significantly to the design and launch of the project, and revised the draft critically for

For peer review only - http://bmjopen.bmj.com/site/about/guidelines.xhtml

intellectual content. AE, EB, UE, MZ and KG contributed to the drafting of the protocol and the continuation of the project. AMB is the coordinator of the study and was primarily responsible for drafting the manuscript and revising it critically for intellectual content.

FUNDING
This work was supported by the Uppsala Region to AS, the Swedish Association of Local Authorities and Regions (SKR) to the department of Obstetrics and Gynecology, Uppsala University Hospital, the Swedish Research Council (Grant number 2020-01965) to AS, as well as the Swedish Brain Foundation and Uppsala University Womher School.

COMPETING INTERESTS
None.

ACKNOWLEDGMENTS
We would like to extend our gratitude to Oskar Burman for creating and continuously adjusting the smartphone research application Mom2B; to Dr. Masoumeh Rezapour, the previous head of department of Obstetrics and Gynecology at Uppsala University Hospital, for support and guidance in the first steps of this project; to Arvid Marklund and Stavros Iliadis for support at the early stages of the app creation; and to Maria Grandahl, Emilia Biskop, and Lisa Lindgren for their valuable input regarding app content and questionnaires.

REFERENCES FOR FIGURE 2
[106-125]

REFERENCES
1. WHO. Maternal Health World Health Organization [Available from: https://www.who.int/health-topics/maternal-health accessed November 2021.
2. Woody CA, Ferrari AJ, Siskind DJ, et al. A systematic review and meta-regression of the prevalence and incidence of perinatal depression. *Journal of Affective Disorders* 2017;219:86-92. doi: 10.1016/J.JAD.2017.05.003
3. WHO. Preterm birth World Health Organization2018 [Available from: https://www.who.int/news-room/fact-sheets/detail/preterm-birth.
4. Association AP. Diagnostic and statistical manual of mental disorders. 5th ed. Washington, DC2013.
5. Moraes GPDA, Lorenzo, L., , Pontes GAR, Montenegro MC, et al. Screening and diagnosing postpartum depression: when and how? *Trends in psychiatry and psychotherapy* 2017;39(1):54-61. doi: 10.1590/2237-6089-2016-0034
6. Bennett HA, Einarson A, Taddio A, et al. Prevalence of depression during pregnancy: Systematic review. *Obstetrics and Gynecology* 2004;103(4):698-709. doi: 10.1097/01.AOG.0000116689.75396.5F

For peer review only - http://bmjopen.bmj.com/site/about/guidelines.xhtml

7. Wikman A, Axfors C, Iliadis SI, et al. Characteristics of women with different perinatal depression trajectories. *Journal of Neuroscience Research* 2020;98(7):1268-82. doi: 10.1002/JNR.24390

8. Field T, Diego M, Hernandez-Reif M. Prenatal depression effects on the fetus and newborn: a review. *Infant Behavior and Development* 2006;29(3):445-55. doi: 10.1016/J.INFBEH.2006.03.003

9. Grote NK, Bridge JA, Gavin AR, et al. A meta-analysis of depression during pregnancy and the risk of preterm birth, low birth weight, and intrauterine growth restriction. *Arch Gen Psychiatry* 2010;67(10):1012-24. doi: 10.1001/archgenpsychiatry.2010.111 [published Online First: 2010/10/06]

10. Kurki T, Hiilesmaa V, Raitasalo R, et al. Depression and anxiety in early pregnancy and risk for preeclampsia. *Obstet Gynecol* 2000;95(4):487-90. doi: 10.1016/s0029-7844(99)00602-x [published Online First: 2000/03/22]

11. Herring SJ, Rose MZ, Skouteris H, et al. Optimizing weight gain in pregnancy to prevent obesity in women and children. *Diabetes Obes Metab* 2012;14(3):195-203. doi: 10.1111/j.1463-1326.2011.01489.x [published Online First: 2011/08/20]

12. Cato K, Sylven SM, Lindbäck J, et al. Risk factors for exclusive breastfeeding lasting less than two months—identifying women in need of targeted breastfeeding support. *PloS one* 2017;12(6):e0179402. doi: https://doi.org/10.1371/journal.pone.0179402

13. Dennis CL, McQueen K. The relationship between infant-feeding outcomes and postpartum depression: a qualitative systematic review. *Pediatrics* 2009;123(4):e736-51. doi: 10.1542/peds.2008-1629 [published Online First: 2009/04/02]

14. Figueiredo B, Canario C, Field T. Breastfeeding is negatively affected by prenatal depression and reduces postpartum depression. *Psychol Med* 2014;44(5):927-36. doi: 10.1017/S0033291713001530 [published Online First: 2013/07/05]

15. Field T. Prenatal Depression Risk Factors, Developmental Effects and Interventions: A Review. *Journal of pregnancy and child health* 2017;4(1) doi: 10.4172/2376-127X.1000301

16. Slomian J, Honvo G, Emonts P, et al. Consequences of maternal postpartum depression: A systematic review of maternal and infant outcomes.

For peer review only - http://bmjopen.bmj.com/site/about/guidelines.xhtml

https://doiorg/101177/1745506519844044 2019;15 doi: 10.1177/1745506519844044

17. Brummelte S, Galea LAM. Postpartum depression: Etiology, treatment and consequences for maternal care. *Hormones and Behavior* 2016;77:153-66. doi: 10.1016/J.YHBEH.2015.08.008
18. Goodman JH. Perinatal depression and infant mental health. *Arch Psychiatr Nurs* 2019;33(3):217-24. doi: 10.1016/j.apnu.2019.01.010 [published Online First: 2019/06/23]
19. Kingston D, Tough S, Whitfield H. Prenatal and postpartum maternal psychological distress and infant development: a systematic review. *Child Psychiatry Hum Dev* 2012;43(5):683-714. doi: 10.1007/s10578-012-0291-4 [published Online First: 2012/03/13]
20. Iliadis SI, Skalkidou A, Ranstrand H, et al. Self-harm thoughts postpartum as a marker for long-term morbidity. *Frontiers in public health* 2018;6:34. doi: https://doi.org/10.3389/fpubh.2018.00034
21. Esscher A, Essen B, Innala E, et al. Suicides during pregnancy and 1 year postpartum in Sweden, 1980-2007. *Br J Psychiatry* 2016;208(5):462-9. doi: 10.1192/bjp.bp.114.161711 [published Online First: 2015/10/24]
22. Lancaster CA, Gold KJ, Flynn HA, et al. Risk factors for depressive symptoms during pregnancy: a systematic review. *Am J Obstet Gynecol* 2010;202(1):5-14. doi: 10.1016/j.ajog.2009.09.007 [published Online First: 2010/01/26]
23. Skalkidou A, Hellgren C, Comasco E, et al. Biological aspects of postpartum depression. *Womens Health (Lond)* 2012;8(6):659-72. doi: 10.2217/whe.12.55 [published Online First: 2012/11/28]
24. O'Hara MW. Postpartum depression: what we know. *Journal of clinical psychology* 2009;65(12):1258-69. doi: https://doi.org/10.1002/jclp.20644
25. Cox EQ, Sowa NA, Meltzer-Brody SE, et al. The perinatal depression treatment cascade: Baby steps toward improving outcomes. 2016;77:1189-200. doi: 10.4088/JCP.15r10174
26. Bränn E, Fransson E, Wikman A, et al. Who do we miss when screening for postpartum depression? A population-based study in a Swedish region. *Journal of Affective Disorders* 2021;287:165-73.

For peer review only

For peer review only - http://bmjopen.bmj.com/site/about/guidelines.xhtml

27. Cox JL, Holden JM, Sagovsky R. Detection of postnatal depression. Development of the 10-item Edinburgh Postnatal Depression Scale. *Br J Psychiatry* 1987;150:782-6. doi: 10.1192/bjp.150.6.782 [published Online First: 1987/06/01]

28. Socialstyrelsen. Vägledning för barnhälsovården, 2014.

29. Massoudi P, Wickberg B, Hwang P. Screening for postnatal depression in Swedish child health care. *Acta Paediatr* 2007;96(6):897-901. doi: 10.1111/j.1651-2227.2007.00292.x [published Online First: 2007/06/01]

30. Yonkers KA, Smith MV, Gotman N, et al. Typical somatic symptoms of pregnancy and their impact on a diagnosis of major depressive disorder. *General hospital psychiatry* 2009;31(4):327-33. doi: 10.1016/j.genhosppsych.2009.03.005

31. Byatt N, Biebel K, Lundquist RS, et al. Patient, provider, and system-level barriers and facilitators to addressing perinatal depression. *Journal of Reproductive and Infant Psychology* 2012;30(5):436-49. doi: https://doi.org/10.1080/02646838.2012.743000

32. Shiffman S, Stone AA, Hufford MR. Ecological momentary assessment. *Annu Rev Clin Psychol* 2008;4:1-32. doi: 10.1146/annurev.clinpsy.3.022806.091415 [published Online First: 2008/05/30]

33. Halfin A. Depression: the benefits of early and appropriate treatment. American Journal of Managed Care. 2007/12/06 ed, 2007:S92-7.

34. Jiménez-Serrano S, Tortajada S, García-Gómez JM. A mobile health application to predict postpartum depression based on machine learning. *Telemedicine and e-Health* 2015;21(7):567-74. doi: 10.1089/tmj.2014.0113

35. Dennis CLE, Janssen PA, Singer J. Identifying women at-risk for postpartum depression in the immediate postpartum period. *Acta Psychiatrica Scandinavica* 2004;110(5):338-46. doi: 10.1111/J.1600-0447.2004.00337.X

36. Ruyak SL, Lowe NK, Corwin EJ, et al. Prepregnancy Obesity and a Biobehavioral Predictive Model for Postpartum Depression. *Journal of Obstetric, Gynecologic & Neonatal Nursing* 2016;45(3):326-38. doi: 10.1016/J.JOGN.2015.12.013

37. Zhang W, Liu H, Silenzio VMB, et al. Machine Learning Models for the Prediction of Postpartum Depression: Application and Comparison Based on a Cohort

For peer review only - http://bmjopen.bmj.com/site/about/guidelines.xhtml

Study. *JMIR Med Inform* 2020;8(4):e15516. doi: 10.2196/15516 [published Online First: 2020/05/01]

38. Cooper PJ, Murray L, Hooper R, et al. The development and validation of a predictive index for postpartum depression. *Psychological Medicine* 1996;26(3):627-34. doi: 10.1017/S0033291700035698
39. Andersson S, Bathula DR, Iliadis SI, et al. Predicting women with depressive symptoms postpartum with machine learning methods. *Scientific Reports 2021 11:1* 2021;11(1):1-15. doi: 10.1038/s41598-021-86368-y
40. De Choudhury M, Counts S, Horvitz E. Predicting postpartum changes in emotion and behavior via social media. *Conference on Human Factors in Computing Systems - Proceedings* 2013:3267-76. doi: 10.1145/2470654.2466447
41. De Choudhury M, Counts S, Horvitz EJ, et al. Characterizing and Predicting Postpartum Depression from Shared Facebook Data. *Proceedings of the 17th ACM conference on Computer supported cooperative work & social computing* 2014 doi: 10.1145/2531602
42. Fatima I, Abbasi BUD, Khan S, et al. Prediction of postpartum depression using machine learning techniques from social media text. *Expert Systems* 2019;36(4):e12409-e09. doi: https://doi.org/10.1111/exsy.12409
43. Mehta D, Newport DJ, Frishman G, et al. Early predictive biomarkers for postpartum depression point to a role for estrogen receptor signaling. *Psychol Med* 2014;44(11):2309-22. doi: 10.1017/S0033291713003231 [published Online First: 2014/02/06]
44. Blencowe H, Cousens S, Chou D, et al. Born too soon: the global epidemiology of 15 million preterm births. *Reprod Health* 2013;10 Suppl 1:S2. doi: 10.1186/1742-4755-10-S1-S2 [published Online First: 2014/03/15]
45. Serenius F, Källén K, Blennow M, et al. Neurodevelopmental outcome in extremely preterm infants at 2.5 years after active perinatal care in Sweden. *Jama* 2013;309(17):1810-20. doi: 10.1001/jama.2013.3786
46. Jacobsson B, Pettersson K, Modzelewska D, et al. Förtidsbörd största perinatala problemet. Läkartidningen, 2019.
47. Räisänen S, Gissler M, Saari J, et al. Contribution of risk factors to extremely, very and moderately preterm births–register-based analysis of 1,390,742

For peer review only - http://bmjopen.bmj.com/site/about/guidelines.xhtml

singleton births. *PLoS One* 2013;8(4):e60660. doi: https://doi.org/10.1371/journal.pone.0060660

48. Staneva A, Bogossian F, Pritchard M, et al. The effects of maternal depression, anxiety, and perceived stress during pregnancy on preterm birth: A systematic review. *Women and Birth* 2015;28(3):179-93. doi: 10.1016/J.WOMBI.2015.02.003

49. Goldenberg RL, Culhane JF, Iams JD, et al. Epidemiology and causes of preterm birth. *The lancet* 2008;371(9606):75-84. doi: https://doi.org/10.1016/S0140-6736(08)60074-4

50. Fransson E, Ortenstrand A, Hjelmstedt A. Antenatal depressive symptoms and preterm birth: a prospective study of a Swedish national sample. *Birth* 2011;38(1):10-6. doi: 10.1111/j.1523-536X.2010.00441.x [published Online First: 2011/02/22]

51. Eklundh A, Grunewald C. Handläggning vid hotande förtidsbörd. Läkartidningen. klinik och vetenskap ed, 2011.

52. Health NIo. What are the risk factors for preterm labor and birth? 2019 [Available from: https://www.nichd.nih.gov/health/topics/preterm/conditioninfo/who_risk accessed October, 2021.

53. Christian LM. Psychoneuroimmunology in pregnancy: immune pathways linking stress with maternal health, adverse birth outcomes, and fetal development. *Neurosci Biobehav Rev* 2012;36(1):350-61. doi: 10.1016/j.neubiorev.2011.07.005 [published Online First: 2011/07/27]

54. Medley N, Vogel JP, Care A, et al. Interventions during pregnancy to prevent preterm birth: an overview of Cochrane systematic reviews. *Cochrane Database of Systematic Reviews* 2018(11) doi: https://doi.org/10.1002/14651858.CD012505.pub2

55. Ferrero DM, Larson J, Jacobsson B, et al. Cross-country individual participant analysis of 4.1 million singleton births in 5 countries with very high human development index confirms known associations but provides no biologic explanation for 2/3 of all preterm births. *PloS one* 2016;11(9):e0162506. doi: https://doi.org/10.1371/journal.pone.0162506

56. Jacobsson B. Prediktion, prevention och behandlingsmetoder. Läkartidningen, 2019.

For peer review only - http://bmjopen.bmj.com/site/about/guidelines.xhtml

57. Varli IH, Wollmann CL, Sandström A. Hotande förtidsbörd/prematurbörd/för tidig födsel: Internetmedicin.se; 2019 [Available from: https://www.internetmedicin.se/behandlingsoversikter/gynekologi-obstetrik/prematura-sammandragningar/.
58. Torous J, Kiang MV, Lorme J, et al. New Tools for New Research in Psychiatry: A Scalable and Customizable Platform to Empower Data Driven Smartphone Research. *JMIR Mental Health* 2016;3(2):e16. doi: 10.2196/mental.5165
59. Meyerowitz-Katz G, Ravi S, Arnolda L, et al. Rates of Attrition and Dropout in App-Based Interventions for Chronic Disease: Systematic Review and Meta-Analysis. *Journal of Medical Internet Research* 2020;22(9):e20283. doi: 10.2196/20283 [published Online First: 2020/09/30]
60. Press G. 12 Big Data Definitions: What's Yours? Forbes, 2014.
61. Pastorino R, De Vito C, Migliara G, et al. Benefits and challenges of Big Data in healthcare: An overview of the European initiatives. *European Journal of Public Health* 2019;29(Supplement_3):23-27. doi: 10.1093/eurpub/ckz168
62. Torous J, Staples P, Onnela JP. Realizing the potential of mobile mental health: new methods for new data in psychiatry. *Curr Psychiatry Rep* 2015;17(8):602. doi: 10.1007/s11920-015-0602-0 [published Online First: 2015/06/16]
63. Olsen J, Melbye M, Olsen SF, et al. The Danish National Birth Cohort: its background, structure and aim. *Scand J Public Health* 2001;29(4):300-7. doi: 10.1177/14034948010290040201 [published Online First: 2002/01/05]
64. Magnus P, Birke C, Vejrup K, et al. Cohort Profile Update: The Norwegian Mother and Child Cohort Study (MoBa). *Int J Epidemiol* 2016;45(2):382-8. doi: 10.1093/ije/dyw029 [published Online First: 2016/04/12]
65. Stoltenberg C, Schjolberg S, Bresnahan M, et al. The Autism Birth Cohort: a paradigm for gene-environment-timing research. *Mol Psychiatry* 2010;15(7):676-80. doi: 10.1038/mp.2009.143 [published Online First: 2010/06/24]
66. Axfors C, Bränn E, Henriksson HE, et al. Cohort profile: The Biology, Affect, Stress, Imaging and Cognition (BASIC) study on perinatal depression in a population-based Swedish cohort. *BMJ Open* 2019;9(10):1-11. doi: 10.1136/bmjopen-2019-031514

For peer review only - http://bmjopen.bmj.com/site/about/guidelines.xhtml

67. Onnela JP, Rauch SL. Harnessing Smartphone-Based Digital Phenotyping to Enhance Behavioral and Mental Health: Nature Publishing Group, 2016:1691-96.

68. Shouval R, Bondi O, Mishan H, et al. Application of machine learning algorithms for clinical predictive modeling: a data-mining approach in SCT. *Bone Marrow Transplant* 2014;49(3):332-7. doi: 10.1038/bmt.2013.146 [published Online First: 2013/10/08]

69. Barnett I, Torous J, Staples P, et al. Relapse prediction in schizophrenia through digital phenotyping: a pilot study. *Neuropsychopharmacology* 2018;43(8):1660-66. doi: 10.1038/s41386-018-0030-z [published Online First: 2018/03/08]

70. Zulueta J, Piscitello A, Rasic M, et al. Predicting Mood Disturbance Severity with Mobile Phone Keystroke Metadata: A BiAffect Digital Phenotyping Study. *J Med Internet Res* 2018;20(7):e241. doi: 10.2196/jmir.9775 [published Online First: 2018/07/22]

71. Hussain-Shamsy N, Shah A, Vigod SN, et al. Mobile Health for Perinatal Depression and Anxiety: Scoping Review. *J Med Internet Res* 2020;22(4):e17011-e11. doi: 10.2196/17011

72. Hahn L, Eickhoff SB, Habel U, et al. Early identification of postpartum depression using demographic, clinical, and digital phenotyping. *Translational psychiatry* 2021;11(1):1-10. doi: https://doi.org/10.1038/s41398-021-01245-6

73. Graham S, Depp C, Lee EE, et al. Artificial Intelligence for Mental Health and Mental Illnesses: an Overview: Current Medicine Group LLC 1, 2019:116-16.

74. Micheli-Tzanakou E. Artificial neural networks: an overview. *Network: Computation in Neural Systems* 2011;22(1-4):208-30. doi: https://doi.org/10.3109/0954898X.2011.638355

75. LeCun Y, Bengio Y, Hinton G. Deep learning. *Nature* 2015;521(7553):436-44. doi: 10.1038/nature14539 [published Online First: 2015/05/29]

76. Miotto R, Wang F, Wang S, et al. Deep learning for healthcare: Review, opportunities and challenges. *Briefings in Bioinformatics* 2018;19(6):1236-46. doi: 10.1093/bib/bbx044

For peer review only - http://bmjopen.bmj.com/site/about/guidelines.xhtml

77. Topol EJ. High-performance medicine: the convergence of human and artificial intelligence. *Nat Med* 2019;25(1):44-56. doi: 10.1038/s41591-018-0300-7 [published Online First: 2019/01/09]

78. Yahata N, Kasai K, Kawato M. Computational neuroscience approach to biomarkers and treatments for mental disorders. *Psychiatry Clin Neurosci* 2017;71(4):215-37. doi: 10.1111/pcn.12502 [published Online First: 2016/12/30]

79. Joshi J, Goecke R, Alghowinem S, et al. Multimodal assistive technologies for depression diagnosis and monitoring. *Journal on Multimodal User Interfaces* 2013;7(3):217-28.

80. SBU. Diagnostik och uppföljning av förstämningssyndrom: en systematisk litteraturöversikt. In: Ekselius L, ed.: Statens beredning för medicinskt utvärdering (SBU), 2012.

81. Levis B, Negeri Z, Sun Y, et al. Accuracy of the Edinburgh Postnatal Depression Scale (EPDS) for screening to detect major depression among pregnant and postpartum women: systematic review and meta-analysis of individual participant data. *bmj* 2020;371 doi: https://doi.org/10.1136/bmj.m4022

82. France DJ, Shiavi RG, Silverman S, et al. Acoustical properties of speech as indicators of depression and suicidal risk. *IEEE transactions on Biomedical Engineering* 2000;47(7):829-37. doi: 10.1109/10.846676

83. Yang Y, Fairbairn C, Cohn JF. Detecting Depression Severity from Vocal Prosody. *IEEE Trans Affect Comput* 2013;4(2):142-50. doi: 10.1109/T-AFFC.2012.38 [published Online First: 2013/04/01]

84. An investigation of depressed speech detection: Features and normalization. Twelfth Annual Conference of the International Speech Communication Association; 2011.

85. On the relative importance of vocal source, system, and prosody in human depression. 2013 IEEE International Conference on Body Sensor Networks; 2013. IEEE.

86. Saeb S, Zhang M, Karr CJ, et al. Mobile Phone Sensor Correlates of Depressive Symptom Severity in Daily-Life Behavior: An Exploratory Study. *J Med Internet Res* 2015;17(7):e175. doi: 10.2196/jmir.4273 [published Online First: 2015/07/17]

For peer review only - http://bmjopen.bmj.com/site/about/guidelines.xhtml

87. Trajectories of depression: unobtrusive monitoring of depressive states by means of smartphone mobility traces analysis. Proceedings of the 2015 ACM international joint conference on pervasive and ubiquitous computing; 2015.

88. Cotten SR, Ford G, Ford S, et al. Internet use and depression among older adults. *Computers in human behavior* 2012;28(2):496-99. doi: https://doi.org/10.1016/j.chb.2011.10.021

89. Katikalapudi R, Chellappan S, Montgomery F, et al. Associating internet usage with depressive behavior among college students. *IEEE Technology and Society Magazine* 2012;31(4):73-80. doi: 10.1109/MTS.2012.2225462

90. Rozgonjuk D, Levine JC, Hall BJ, et al. The association between problematic smartphone use, depression and anxiety symptom severity, and objectively measured smartphone use over one week. *Computers in Human Behavior* 2018;87:10-17.

91. Elhai JD, Tiamiyu MF, Weeks JW, et al. Depression and emotion regulation predict objective smartphone use measured over one week. *Personality and Individual Differences* 2018;133:21-28. doi: https://doi.org/10.1016/j.paid.2017.04.051

92. Aledavood T, Torous J, Triana Hoyos AM, et al. Smartphone-Based Tracking of Sleep in Depression, Anxiety, and Psychotic Disorders. *Curr Psychiatry Rep* 2019;21(7):49. doi: 10.1007/s11920-019-1043-y [published Online First: 2019/06/05]

93. DeMasi O, Feygin S, Dembo A, et al. Well-Being Tracking via Smartphone-Measured Activity and Sleep: Cohort Study. *JMIR Mhealth Uhealth* 2017;5(10):e137. doi: 10.2196/mhealth.7820 [published Online First: 2017/10/07]

94. Torous J, Staples P, Barnett I, et al. Characterizing the clinical relevance of digital phenotyping data quality with applications to a cohort with schizophrenia. *NPJ Digit Med* 2018;1:15. doi: 10.1038/s41746-018-0022-8 [published Online First: 2018/04/06]

95. Collins GS, Reitsma JB, Altman DG, et al. Transparent Reporting of a multivariable prediction model for Individual Prognosis Or Diagnosis (TRIPOD): the TRIPOD Statement. *Br J Surg* 2015;102(3):148-58. doi: 10.1002/bjs.9736 [published Online First: 2015/01/30]

For peer review only - http://bmjopen.bmj.com/site/about/guidelines.xhtml

96. Mehrotra A, Musolesi M. Using autoencoders to automatically extract mobility features for predicting depressive states. *Proceedings of the ACM on Interactive, Mobile, Wearable and Ubiquitous Technologies* 2018;2(3):1-20. doi: https://doi.org/10.1145/3264937
97. Mental health analysis using deep learning for feature extraction. Proceedings of the ACM India Joint International Conference on Data Science and Management of Data; 2018.
98. Saqib K, Khan AF, Butt ZA. Machine Learning Methods for Predicting Postpartum Depression: Scoping Review. *JMIR Ment Health* 2021;8(11):e29838. doi: 10.2196/29838 [published Online First: 2021/11/26]
99. Multimodal measurement of depression using deep learning models. Proceedings of the 7th Annual Workshop on Audio/Visual Emotion Challenge; 2017.
100. Towards multi-modal anticipatory monitoring of depressive states through the analysis of human-smartphone interaction. Proceedings of the 2016 ACM international joint conference on pervasive and ubiquitous computing: adjunct; 2016.
101. Atrey PK, Hossain MA, El Saddik A, et al. Multimodal fusion for multimedia analysis: a survey. *Multimedia systems* 2010;16(6):345-79. doi: 10.1007/s00530-010-0182-0
102. Fransson E, Karalexi M, Kimmel M, et al. Mental health among pregnant women during the pandemic in Sweden, a mixed methods approach using data from the Mom2B mobile application for research. *MedRxiv* 2020 doi: https://doi.org/10.1101/2020.12.18.20248466
103. Sheehan AM, McGee H. Screening for depression in medical research: ethical challenges and recommendations. *BMC Med Ethics* 2013;14:4. doi: 10.1186/1472-6939-14-4 [published Online First: 2013/01/10]
104. Zhou C, Hu H, Wang C, et al. The effectiveness of mHealth interventions on postpartum depression: A systematic review and meta-analysis. *J Telemed Telecare* 2020:1357633X20917816. doi: 10.1177/1357633X20917816 [published Online First: 2020/04/21]
105. Bruno A, Celebre L, Mento C, et al. When fathers begin to falter: a comprehensive review on paternal perinatal depression. *International journal*

For peer review only

of environmental research and public health 2020;17(4):1139. doi: https://doi.org/10.3390/ijerph17041139

106. Topp CW, Ostergaard SD, Sondergaard S, et al. The WHO-5 Well-Being Index: a systematic review of the literature. *Psychother Psychosom* 2015;84(3):167-76. doi: 10.1159/000376585 [published Online First: 2015/04/04]

107. Zigmond AS, Snaith RP. The hospital anxiety and depression scale. *Acta Psychiatr Scand* 1983;67(6):361-70. doi: 10.1111/j.1600-0447.1983.tb09716.x [published Online First: 1983/06/01]

108. Eklund M, Backstrom M, Tuvesson H. Psychometric properties and factor structure of the Swedish version of the Perceived Stress Scale. *Nord J Psychiatry* 2014;68(7):494-9. doi: 10.3109/08039488.2013.877072 [published Online First: 2014/01/28]

109. Rosengren A, Orth-Gomer K, Wedel H, et al. Stressful life events, social support, and mortality in men born in 1933. *BMJ* 1993;307(6912):1102-5. doi: 10.1136/bmj.307.6912.1102 [published Online First: 1993/10/30]

110. Greenwald R, Rubin A. Assessment of posttraumatic symptoms in children: Development and preliminary validation of parent and child scales. *Research on Social Work Practice* 1999;9(1):61-75. doi: https://doi.org/10.1177/104973159900900105

111. Hildingsson I, Rubertsson C, Karlstrom A, et al. Exploring the Fear of Birth Scale in a mixed population of women of childbearing age-A Swedish pilot study. *Women Birth* 2018;31(5):407-13. doi: 10.1016/j.wombi.2017.12.005

112. Craig CL, Marshall AL, Sjostrom M, et al. International physical activity questionnaire: 12-country reliability and validity. *Med Sci Sports Exerc* 2003;35(8):1381-95. doi: 10.1249/01.MSS.0000078924.61453.FB

113. Rosen R, Brown C, Heiman J, et al. The Female Sexual Function Index (FSFI): a multidimensional self-report instrument for the assessment of female sexual function. *J Sex Marital Ther* 2000;26(2):191-208. doi: 10.1080/009262300278597 [published Online First: 2000/04/27]

114. Bastien CH, Vallieres A, Morin CM. Validation of the Insomnia Severity Index as an outcome measure for insomnia research. *Sleep Med* 2001;2(4):297-307. doi: 10.1016/s1389-9457(00)00065-4 [published Online First: 2001/07/05]

For peer review only - http://bmjopen.bmj.com/site/about/guidelines.xhtml

115. Wagnild GM, Young HM. Development and psychometric evaluation of the Resilience Scale. *J Nurs Meas* 1993;1(2):165-78. [published Online First: 1993/01/01]

116. Eriksson M, Lindstrom B. Validity of Antonovsky's sense of coherence scale: a systematic review. *J Epidemiol Community Health* 2005;59(6):460-6. doi: 10.1136/jech.2003.018085

117. Dennis C, Boyce P. Further psychometric testing of a brief personality scale to measure vulnerability to postpartum depression. *Journal of Psychosomatic Obstetrics & Gynecology* 2004;25(3-4):305-11. doi: https://doi.org/10.1080/01674820400017962

118. Brennan KA, Clark CL, Shaver P. Self-report measures of adult romantic attachment. *Attachment theory and close relationships* 1998:46-76.

119. Burman M, Norlander A-K, Carlbring P, et al. Närmare varandra: Nio veckor till en starkare parrelation: Natur & Kultur 2018.

120. Morelius E, Elander A, Saghamre E. A Swedish translation and validation of the Mother-to-Infant Bonding Scale. *Scand J Public Health* 2021;49(4):465-70. doi: 10.1177/1403494820910336 [published Online First: 2020/03/12]

121. Brockington IF, Fraser C, Wilson D. The Postpartum Bonding Questionnaire: a validation. *Arch Womens Ment Health* 2006;9(5):233-42. doi: 10.1007/s00737-006-0132-1

122. Putnam SP, Helbig AL, Gartstein MA, et al. Development and assessment of short and very short forms of the Infant Behavior Questionnaire–Revised. *Journal of personality assessment* 2014;96(4):445-58.

123. Östberg M, Hagekull B. A structural modeling approach to the understanding of parenting stress. *J Clin Child Psychol* 2000;29(4):615-25. doi: 10.1207/S15374424JCCP2904_13 [published Online First: 2000/12/29]

124. Barrett G, Smith SC, Wellings K. Conceptualisation, development, and evaluation of a measure of unplanned pregnancy. *J Epidemiol Community Health* 2004;58(5):426-33. doi: 10.1136/jech.2003.014787 [published Online First: 2004/04/15]

125. Bates JE, Freeland CA, Lounsbury ML. Measurement of infant difficultness. *Child Dev* 1979;50(3):794-803. doi: https://doi.org/10.2307/1128946

For peer review only - http://bmjopen.bmj.com/site/about/guidelines.xhtml

LEGENDS FOR FIGURES

Figure 1. *From top to bottom, the gray content blocks in the main column represent installed apps (downloads of the Mom2B app by unique users from either app store (iOS) or google play (Android), registered users (individuals that have submitted registration information in the app), signed consents (registered users that have consented to contributing data, and signed these consents electronically), and finally, data (participants with signed consents that, at minimum, have completed the EPDS[27] at least once. The latter two blocks also illustrate the signed consents and available data, respectively, by type of data (survey, voice and passive data). The intersections of the Venn diagrams are non-exclusive, meaning that the number count in the intersection of surveys and passive data, for example, can include individuals who have also contributed voice-recordings. This flowchart reflects data last downloaded on September 6, 2021.*

Figure 2. *Timeline of validated instruments administered in the Mom2B study during pregnancy and postpartum periods, and the number of occurences for each instrument throughout the study period. Surveys become available to users for varying periods of time and will disappear once completed or when their period of availability is over. Note: EPDS, Edinburgh Postnatal Depression Scale[27]; WHO-5, World Health Organization wellbeing index[106]; DSM-screening, Diagnostic and Statistical Manual, 5th edition, criterion for depression; DSM Screening – short is a shortened version of the DSM Screening with selected questions chosen by the research team; HADS, Hospital Anxiety and Depression Scale[107]; PSS, Perceived stress scale[108]; SLE, Stressful life events[109]; LITE, Lifetime Influence of Traumatic Experiences[110]; FOBS, Fear of birth[111]; IPAQ, International physical activity questionnaire[112]; FSFI, Female sexual function index[113]; ISI, Insomnia Severity Index[114]; RS-14, Resilience scale[115]; SOC, Sense of coherence[116]; VPSQ, Vulnerability personality style questionnaire[117]; ECR, Experience in Close Relationships scale[118]; Valentin scale (relationship with your partner)[119]; S-MIBS, Swedish Mother to Infant Bonding Scale[120]; PBQ, Postpartum Bonding Questionnaire[121]; IBQ, Infant Behavior Questionnaire[122]; SPSQ, Swedish Parenthood Stress Questionnaire[123]; LMUP, London Measure of Unplanned Pregnancy[124]; ICQ, Infant Characteristics Questionnaire[125].*

Figure 3. *Timeline of self-developed surveys administered in the Mom2B study during pregnancy and postpartum periods, and the number of occurences for each instrument throughout the study period. Surveys become available to users for varying periods of time and will disappear once completed or when their period of availability is over.*

Figure 4. *Flow of data from user to servers for storage and analysis. Data passes through secure servers accessible only by authorized members of the Mom2B team, and can be decrypted for analysis in Bianca when needed.*

Figure 5. *A multimodal ML model for PND diagnosis. The extracted features can be classified into three categories: acoustic signals, time-series features and categorical features. We can then determine the most suitable model for each category. For example, for acoustic signals, we would apply Convolutional Neural Network; for time-series data, we would apply recurrent neural network such as long short memory; and for numerical variables, we would apply DNNs such as transformers, or traditional models like extremely randomized trees, gradient boost trees, etc. These models can yield high-dimension representations of multimodal features. After feature fusion, the integrated features will be fed in to another neural network for prediction.*

For peer review only

For peer review only - http://bmjopen.bmj.com/site/about/guidelines.xhtml

For peer review only

For peer review only - http://bmjopen.bmj.com/site/about/guidelines.xhtml

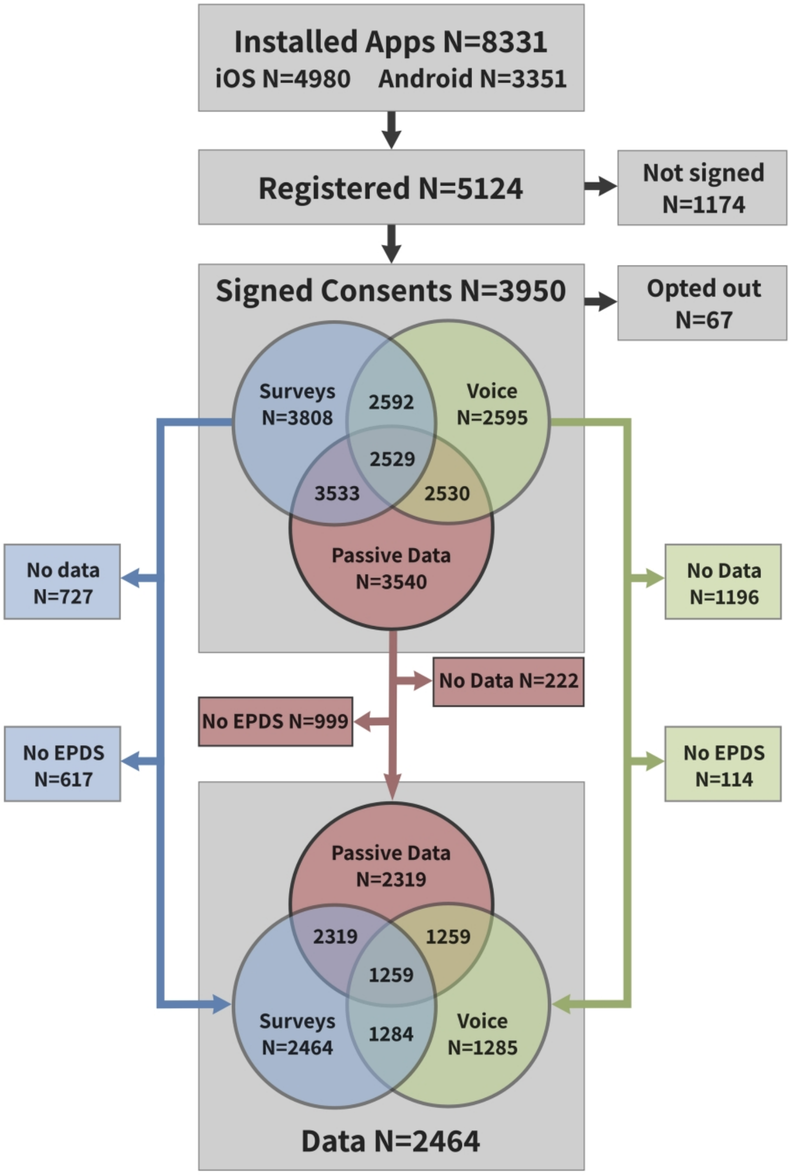

From top to bottom, the gray content blocks in the main column represent installed apps (downloads of the Mom2B app by unique users from either app store (iOS) or google play (Android), registered users (individuals that have submitted registration information in the app), signed consents (registered users that have consented to contributing data, and signed these consents electronically), and finally, data (participants with signed consents that, at minimum, have completed the EPDS[27] at least once. The latter two blocks also illustrate the signed consents and available data, respectively, by type of data (survey, voice and passive data). The intersections of the Venn diagrams are non-exclusive, meaning that the number count in the intersection of surveys and passive data, for example, can include individuals who have also contributed voice-recordings. This flowchart reflects data last downloaded on September 6, 2021.

105x144mm (600 x 600 DPI)

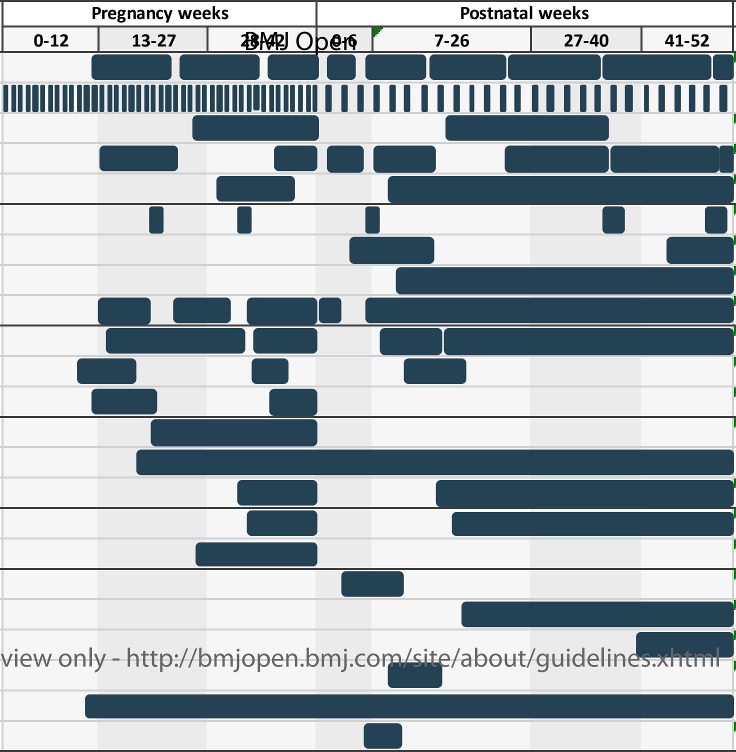

	Validated surveys	Frequency: Pregnancy	Frequency: Postnatal
Mental health	EPDS	3	6
	WHO-5	42 (weekly)	26 (biweekly)
	DSM-screening	1	1
	DSM-screening short	2	5
	HADS	1	1
Stress	PSS	2	3
	SLE	0	2
	LITE	0	1
	FOBS	3	2
General Health	IPAQ	2	2
	FSFI	2	1
	ISI	2	0
Personality	RS-14	1	0
	SOC	Baseline	
	VPSQ	1	1
Attachment in adult relationships	ECRS	1	1
	Valentine Scale	1	0
Parenthood	S-MIBS	0	1
	PBQ	0	1
	IBQ	0	1
	SPSQ	0	1
	LMUP	Baseline	
	ICQ	0	1

For peer review only - http://bmjopen.bmj.com/site/about/guidelines.xhtml

	Self-developed surveys	Frequency: Pregnancy	Frequency: Postnatal
History	Sociodemographic info	Baseline	
	Psychiatric history	Baseline	
	Medical history	Baseline	
	Gynecological health and history	Baseline	
	Physical activity before pregnancy	Baseline	
	Lifestyle before preg	Baseline	
General health	Weight	9	4
	Sleep	4	4
	Pregnancy and postpartum complications	3	5
	Diet	2	1
	Medications	1	1
Psychological health	Stress level	8	0
	Mood	2	2
	Substance abuse	1	1
	Ongoing psychological treatment	0	2
	Recent stressful life events	2	1
Lifestyle	Perinatal physical activity	2	2
	Daily routine and leisure activities	1	6
Perinatal experiences and lifestyle	Neonatal care	0	1
	Impact of coronavirus	3	2
	Breastfeeding experience	1	5
Relationships	Violence in close relationships	1	0
	Social support	3	2
	Co-parenting experience	0	1

For peer review only - http://bmjopen.bmj.com/site/about/guidelines.xhtml

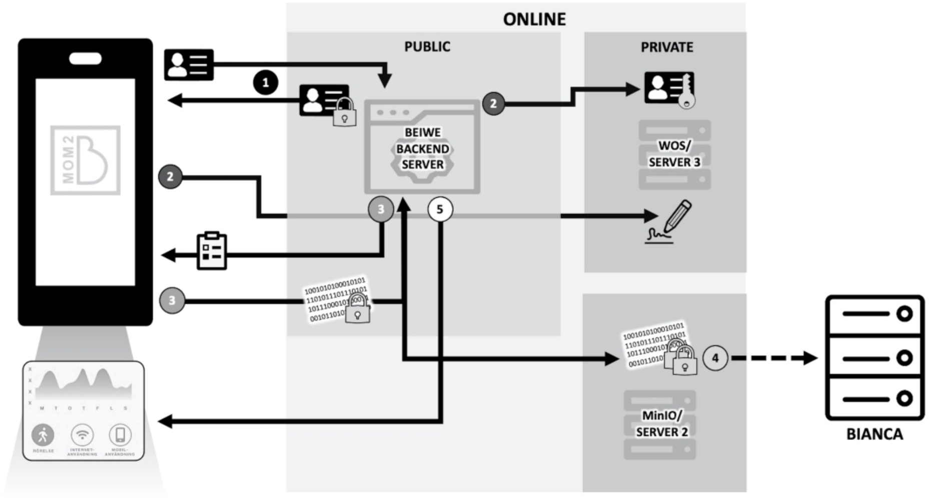

Flow of data from user to servers for storage and analysis. Data passes through secure servers accessible only by authorized members of the Mom2B team, and can be decrypted for analysis in Bianca when needed.

639x319mm (300 x 300 DPI)

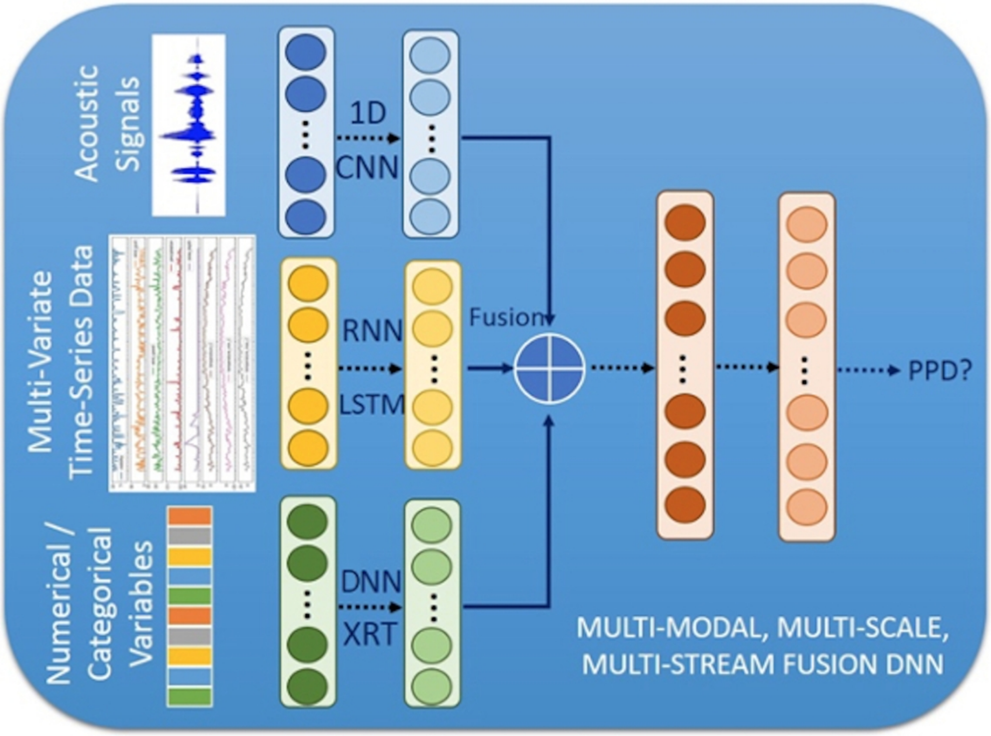

A multimodal ML model for PND diagnosis. The extracted features can be classified into three categories: acoustic signals, time-series features and categorical features. We can then determine the most suitable model for each category. For example, for acoustic signals, we would apply Convolutional Neural Network; for time-series data, we would apply recurrent neural network such as long short memory; and for numerical variables, we would apply DNNs such as transformers, or traditional models like extremely randomized trees, gradient boost trees, etc. These models can yield high-dimension representations of multimodal features. After feature fusion, the integrated features will be fed in to another neural network for prediction.

210x155mm (300 x 300 DPI)

Translated English version of participant information and consent form given digitally to participants via the Mom2B app. See further below for original Swedish version.

PARTICIPANT INFORMATION MOM2B

We want to ask you if you want to participate in a research project. Now you will receive information about the project and what it means to participate. There is a lot of text to read, but the text can also be read again in the app.

At the end of the information, you will have to give consent to the study. You can go into settings at any time and change your consents.

If you have questions about the study, you are welcome to contact us by e-mail: mom2b@kbh.uu.se.

WHAT IS THE PROJECT AND WHY DO YOU WANT ME TO PARTICIPATE?

Postpartum depression is when women experience depression during pregnancy and up to one year after the baby is born. About 12% of all mothers experience postpartum depression. This can lead to suffering and problems for the affected mother and her children. There are effective treatment options. If research finds better ways to identify women at high risk for postpartum depression, more people could receive care early.

During pregnancy and childbirth, the mother and baby can have complications. An example is premature birth, when the baby is born before 37 weeks of pregnancy. Children born prematurely are at greater risk of becoming ill as newborns. In Sweden, about 5% of all births are premature. If the research provides better knowledge to predict which women are at higher risk of being affected, some complications could perhaps be prevented.

With the help of smartphones, it is possible to collect information about people's everyday lives. Examples of measurements are how much a person moves and how active they are on social media. Such measurements have in previous studies been able to predict mental state, but it has not been studied in pregnant women or new mothers. No one has studied whether the measurements can predict complications during pregnancy and childbirth.

THE PURPOSE OF THIS STUDY

The purpose is to use data collected through this app, MOM2B, to improve the detection of women who are at higher risk of developing mental or physical illness during pregnancy and childbirth.

HOW DOES THE STUDY WORK?

You can participate in the study if these three points apply to you:

• I am pregnant or have given birth less than three months ago.
• I am currently using my own smartphone.
• I am at least 18 years old.

At the end of this information section, you can choose which parts of the study you wish to participate in. As a participant, you can choose to contribute:

• To answer questions via this app about your health, lifestyle and background as well as about the child. The questions are compiled by the research team and examine possible risk factors. Questions are sent out approximately 1-2 times per week and usually contain about 5 questions, a maximum of about 15 questions.

For peer review only

• Information about your general mobile use. This means how much internet you use, how much of the mobile processor you use, how often you have access to the internet and how often the charger is connected. The information is collected passively without you having to enter it.
• Information on how much you move. Then the phone's GPS and accelerometer are used. We do not save any specific GPS coordinates, but only information about how you moved in relation to a randomly selected location (eg 100m north, then 50m east, etc.). The information is collected passively without you having to enter it.
• Information about how active you are on social media. What is measured is the number of times someone "likes" something you posted, the number of times you make a status update and the number of times you send a message. Only the number of activities is saved, not the content. What you like or what text you wrote to whom is not saved. The information is collected passively without you having to enter it. The social media that is included right now is Facebook. There may be more (such as Instagram, LinkedIn, Twitter and Snapchat) but then you will get a new question about participation for these.
• To record the sound of your voice when reading texts or numbers. With the help of these audio recordings, we examine e.g. speech tempo and voice mode.
• Information from national health registers and care quality registers about your health, lifestyle and background from one year before the birth to 15 years after the birth. Examples of information that can be obtained are diagnoses you receive in contact with healthcare, level of education, income and employment. The information is collected via the National Board of Health and Welfare and Statistics Sweden.

POSSIBLE CONSEQUENCES AND RISKS OF PARTICIPATING IN THIS STUDY
Answering questions about mental health can involve a risk of emotional strain as some questions may be perceived as sensitive. You choose which questions you want to answer and if you need support, you can contact us for guidance.
If your questionnaire responses indicate severe symptoms of depression, you will receive an automatic message with information on how to seek care. Because your answers are coded and encrypted, we cannot contact you through the app, but you will have the opportunity to enter your phone number so that we can call you during office hours.
Although the research group takes measures to achieve very high data security (such as storing data under code instead of identity, encrypting data directly when collecting and storing data on Uppsala University's servers with a high level of physical and electronic security), it is impossible to completely rule out data intrusion. If data breach is suspected, it will be handled in accordance with current legislation.

ARE THERE ANY BENEFITS?
As a participant, you get access to a knowledge bank in the MOM2B app about health during pregnancy and after childbirth. You also get the opportunity to submit proposals for new topics to the knowledge bank. In addition, participation will not provide any immediate benefits.

WHAT HAPPENS TO MY TASKS?
The project will collect and register information about you. Here we describe how we protect your information.

For peer review only

Code number
You will automatically receive a code number so that no information is stored together with your identity. When we analyze the data and report the results, you will remain anonymous. Responses to questionnaires will be presented so that individuals' responses cannot be traced. The link between your code number and your identity is stored in an isolated location, separate from the information collected.

Encryption
Immediately when data is collected, an encryption is performed. This means that the information is made illegible. Only with the encryption key will the information be readable. All transport of data over networks is done with an additional encryption. The encryption key is stored on a storage unit without a network connection, which is stored locked in a safe at Uppsala University.

Storage of data
The collected information is stored coded and encrypted on a server at Uppsala University indefinitely. Prior to our analyses, the encryption will be unlocked, but the information will remain coded. Only researchers in our research group will have access to the data. Our main analysis, so-called machine learning, is done on computers in a locked space at Uppsala University that has no network connection. All in all, this means that the risk of data intrusion is greatly minimized.
You can choose whether some of your survey answers are to be presented in simplified form on your smartphone under "Statistics". No other data is stored on the phone itself. Identifiable personal data will never be shared with any third party other than the administrators at the National Board of Health and Welfare, Statistics Sweden and the quality of care register, who retrieve information from health registers for the participants who have consented to this. Immediately after the link, all data is deidentified again by coding.
Your answers and your results will be processed so that unauthorized persons cannot take part in them. Uppsala University is responsible for your personal data. According to the EU Data Protection Regulation, you have the right to access the information about you that is handled in the study free of charge and, if necessary, have any errors corrected. You can also request that information about you be deleted and that the processing of your personal data be restricted. If you want to take part in the information, you should contact the principal researcher, Alkistis Skalkidou, telephone: 070 167 97 78 or e-mail: mom2b@kbh.uu.se. The Data Protection Officer can be reached by e-mail: dataskyddsombud@uu.se. If you are dissatisfied with how your personal data is processed, you have the right to report this to the Swedish Data Inspectorate, which is the supervisory authority. The answers to surveys will be compiled statistically in anonymous form and presented so that individuals' answers cannot be traced.

How do I get information about the results of the study?
Results from the project will be presented on our website https://www.mom2b.se/, in scientific articles and at national and international conferences. The results of the study will present the participants' answers in groups and your individual answers will not be traceable to you.

For peer review only

Insurance and compensation
As a participant in this study, you are covered by the patient injury association. No compensation will be given for participating in the study.

PARTICIPATION IS VOLUNTARY
Your participation is voluntary and you can choose to cancel your participation at any time. If you choose not to participate or want to cancel your participation, you do not have to state why, nor will it affect your future care or treatment.
If you would like to cancel your participation, please contact the lead researcher, Alkistis Skalkidou (see below). You are also welcome to contact us at mom2b@kbh.uu.se if you have questions about the study.

Responsible for the study are:
Professor Alkistis Skalkidou
Department of Women's and Children's Health
Akademiska Sjukhuset, 751 85 Uppsala

Email: mom2b@kbh.uu.se
Phone: 070 167 97 78

STEP 1: CONSENT
I have received written information about the study and have had the opportunity to ask questions. I have access to the written participant information electronically in the MOM2B smartphone application at any time.
In the paragraphs that come now, you decide which parts of the study you want to participate in by clicking in the boxes for Yes or No.
I approve of my participation in the study. (You must answer 'Yes' to be able to participate in the study.)
□ Yes □ No.

STEP 2: SURVEYS
I agree to answer questions about my health, lifestyle and background as well as the child's health.
□ Yes □ No.

STEP 3: MOBILE USE
I agree that data about my general mobile use is stored. This means internet consumption (kb/s), processor usage (CPU%/s), internet access and whether my phone is charging or not.
□ Yes □ No.

STEP 4: MOBILITY PATTERN
I agree that data is stored about how much I move. We want to record how much and how fast you move, but not exactly where. Only location data from an unknown point is saved, not your exact location. If you select "no", no location data, relative or exact, is saved.
□ Yes □ No.

STEP 5: SOCIAL MEDIA

For peer review only

I agree that information about how active I am on social media is stored. This means the number of times I perform an activity (that someone likes one of your status updates or that you post a status update) but not the text or image content of the activity. Therefore, this does NOT mean information about who is contacted, what is written or what is liked.
□ Yes □ No.

STEP 6: AUDIO RECORDING
I agree to record the sound of my voice when I read out predetermined texts and numbers.
□ Yes □ No.

STEP 7: HEALTH REGISTERS
I agree that information is obtained from national health registers and care quality registers about my health, lifestyle and background from one year before to 15 years after the birth. Examples are diagnoses I get in contact with healthcare, level of education, income and employment.
□ Yes □ No.

STEP 8: PARTICIPATION IN STUDIES
To improve the app and increase the possibilities for predicting postpartum depression and facilitate other studies in women's health, we would like to ask you if we can contact you at a later opportunity with a request for participation in other studies.
At that time, you always have the opportunity to decline the offer of participation. Participation in other studies is completely voluntary and if you choose not to be contacted, you will not be negatively affected in any way. Select one or both of the following options to be contacted:
□ I wish to be contacted for inquiries about participation in sub-studies within Mom2B
□ I wish to be contacted for inquiries about participation in other relevant studies on women's health

SIGNATURE
To be able to use the app, we need your signature
First and last name
Click here to sign

For peer review only

Original Swedish version of participant information and consent form given digitally to participants via the Mom2B app.

DELTAGARINFORMATION MOM2B
Vi vill fråga dig om du vill delta i ett forskningsprojekt. Nu kommer du att få information om projektet och vad det innebär att delta. Det är mycket text att läsa men texten finns också att läsa igen i appen.
I slutet av informationen kommer du få lämna samtycke till studien. Du kan när som helst gå in i inställningar och ändra dina samtycken.
Om du har frågor om studien är du välkommen att kontakta oss på e post: mom2b@kbh.uu.se.

For peer review only

VAD ÄR DET FÖR PROJEKT OCH VARFÖR VILL NI ATT JAG SKA DELTA?
Förlossningsdepression kallas det när kvinnor drabbas av nedstämdhet under graviditeten och upp till ett år efter att barnet fötts. Ungefär 12% av alla mammor får förlossningsdepression. Detta kan leda till lidande och problem för den drabbade mamman och hennes barn. Det finns effektiva behandlingsalternativ. Om forskningen hittar bättre sätt att identifiera kvinnor med hög risk för förlossningsdepression skulle fler kunna få vård tidigt.
Under graviditeten och vid förlossningen kan mamman och barnet råka ut för komplikationer. Ett exempel är för tidig födsel, när barnet föds innan 37 graviditetsveckor. Barn som föds för tidigt har större risk för att bli sjuka som nyfödda. I Sverige är cirka 5% av alla födslar för tidiga. Om forskningen ger bättre kunskap för att förutsäga vilka kvinnor som har högre risk att drabbas skulle en del komplikationer kanske kunna förhindras.
Med hjälp av smartphones går det att samla in information om människors vardagsliv. Exempel på mätningar är hur mycket en person förflyttar sig och hur aktiv den är på sociala medier. Sådana mätningar har i tidigare studier kunnat förutsäga psykiskt mående, men det har inte studerats hos gravida kvinnor eller nyblivna mammor. Ingen har heller studerat om mätningarna kan förutsäga komplikationer under graviditeten och vid förlossningen.

SYFTET MED STUDIEN
Syftet är att använda data som samlas via den här appen, MOM2B, för att förbättra upptäckandet av kvinnor som har en högre risk att drabbas av psykisk eller fysisk sjuklighet under graviditet och förlossning.

HUR GÅR STUDIEN TILL?
Du kan delta i studien om dessa tre punkter stämmer in på dig:
- Jag är gravid eller har fött barn för mindre än tre månader sedan.
- Jag använder just nu min egen smartphone.
- Jag är minst 18 år gammal.

I slutet av det här informationsavsnittet kan du välja vilka delar av studien du önskar delta i.
Som deltagare kan du välja att bidra med:
- Att svara på frågor via den här appen om din hälsa, livsstil och bakgrund samt om barnet. Frågorna är sammanställda av forskarteamet och undersöker möjliga riskfaktorer. Frågor skickas ut ungefär 1-2 gånger per vecka och innehåller som regel ca 5 frågor, max ca 15 frågor.

- Uppgifter om din allmänna mobilanvändning. Med detta menas hur mycket internet du använder, hur mycket av mobilens processor du använder, hur ofta du har tillgång till internet och hur ofta laddaren är inkopplad. Informationen samlas passivt utan att du behöver skriva in den.
- Uppgifter om hur mycket du förflyttar dig. Då används telefonens GPS och accelerometer. Vi sparar inte några specifika GPS-koordinater, utan bara information om hur du rört dig i förhållande till en slumpmässigt utvald plats (t.ex. 100m norrut, sen 50m österut, osv.). Informationen samlas passivt utan att du behöver skriva in den.
- Uppgifter om hur aktiv du är på sociala medier. Det som mäts är antalet gånger någon ”gillar” något du postat, antalet gånger du gör en statusuppdatering och antalet gånger du skickar ett meddelande. Bara antalet aktiviteter sparas, inte innehållet. Vad du gillar eller vilken text du skrivit till vem sparas inte. Informationen samlas passivt utan att du behöver skriva in den. De sociala medier som ingår just nu är Facebook. Det kan komma att bli fler (så som Instagram, LinkedIn, Twitter och Snapchat) men då kommer du att få en ny fråga om deltagande för dessa.
- Att spela in ljudet av din röst när du läser upp texter eller siffror. Med hjälp av dessa ljudinspelningar undersöker vi t.ex. taltempo och röstläge.
- Uppgifter från nationella hälsoregister och vårdkvalitetsregister om din hälsa, livsstil och bakgrund från ett år före förlossningen till 15 år efter förlossningen. Exempel på information som kan inhämtas är diagnoser du får i kontakt med vården, utbildningsnivå, inkomst och sysselsättning. Uppgifterna hämtas via Socialstyrelsen och Statistiska Centralbyrån.

MÖJLIGA FÖLJDER OCH RISKER MED ATT DELTA I STUDIEN
Att besvara frågor om psykisk hälsa kan innebära en risk för känslomässig påfrestning eftersom vissa frågor kan uppfattas som känsliga. Du väljer själv vilka frågor du vill svara på och ifall du behöver stöd kan du kontakta oss för vägledning.
Om dina enkätsvar skulle tyda på allvarliga symptom på nedstämdhet kommer du att få ett automatiskt meddelande med information om hur du kan söka vård. Eftersom dina svar är kodade och krypterade kan vi inte kontakta dig genom appen, men du kommer att ha möjlighet att ange ditt telefonnummer så att vi kan ringa upp dig under kontorstid.

Trots att forskargruppen vidtar åtgärder för att uppnå mycket hög datasäkerhet (som att lagra data under kod istället för identitet, kryptera data direkt vid insamling och förvara data på Uppsala Universitets servrar med hög nivå av fysisk och elektronisk säkerhet) är det omöjligt att helt utesluta dataintrång. Om dataintrång mot förmodan skulle ske kommer det att hanteras enligt gällande lagstiftning.

FINNS DET NÅGRA FÖRDELAR?

Du som deltagare får tillgång till en kunskapsbank i MOM2B-appen om hälsa under graviditet och efter förlossning. Du får också möjlighet att skicka in förslag på nya ämnen till kunskapsbanken. Utöver detta kommer deltagandet inte ge några omedelbara fördelar.

VAD HÄNDER MED MINA UPPGIFTER?

For peer review only

Projektet kommer att samla in och registrera information om dig. Här beskriver vi hur vi skyddar dina uppgifter.

Kodnummer

Du får automatiskt ett kodnummer så att inga uppgifter lagras tillsammans med din identitet. När vi analyserar uppgifterna och redovisar resultaten kommer du att förbli anonym. Svar på enkäter kommer att presenteras så att enskilda personers svar inte kan spåras. Kopplingen mellan ditt kodnummer och din identitet lagras på en isolerad plats, separat från de insamlade uppgifterna.

Kryptering

Direkt när uppgifter samlas in görs en kryptering. Detta betyder att informationen görs oläslig. Bara med krypteringsnyckeln blir uppgifterna läsliga. Alla transporter av data över nätverk görs med ytterligare en kryptering. Krypteringsnyckeln sparas på en lagringsenhet utan nätverkskoppling, som förvaras inlåst i ett kassaskåp på Uppsala Universitet.

Förvaring av datauppgifter

De insamlade uppgifterna sparas kodade och krypterade på en server vid Uppsala Universitet på obestämd tid. Inför våra analyser kommer krypteringen att låsas upp, men uppgifterna förblir kodade. Bara forskare i vår forskargrupp kommer att ha tillgång till uppgifterna. Vår huvudsakliga analys, s.k. maskininlärning, görs på datorer i ett låst utrymme på Uppsala Universitet som inte har någon nätverksuppkoppling. Detta innebär sammantaget att risken för dataintrång kraftigt minimeras.

Du kan välja ifall vissa av dina enkätsvar ska presenteras i förenklad form på din smartphone under ”Statistik”. Inga andra uppgifter lagras på själva telefonen.

Identifierbara personuppgifter kommer aldrig att delas med någon tredje part förutom de handläggare på Socialstyrelsen, Statistiska Centralbyrån och vårdkvalitetsregister som hämtar information från hälsoregister för de deltagare som samtyckt till detta. Omedelbart efter sammanlänkningen avidentifieras all data igen genom kodning.

Dina svar och dina resultat kommer att behandlas så att inte obehöriga kan ta del av dem. Ansvar för dina personuppgifter har Uppsala Universitet. Enligt EU:s dataskyddsförordning har du rätt att kostnadsfritt få ta del av de uppgifter om dig som hanteras i studien och vid behov få eventuella fel rättade. Du kan också begära att uppgifter om dig raderas samt att behandlingen av dina personuppgifter begränsas. Om du vill ta del av uppgifterna ska du kontakta huvudansvarig forskare, Alkistis Skalkidou, telefon: 070 167 97 78 eller e-post: mom2b@kbh.uu.se. Dataskyddsombud nås på e-post: dataskyddsombud@uu.se. Om du är missnöjd med hur dina personuppgifter behandlas har du rätt att anmäla detta till Datainspektionen, som är tillsynsmyndighet. Svaren på enkäter kommer att sammanställas statistiskt i anonym form och presenteras så att enskilda personers svar inte kan spåras.

Hur får jag information om resultatet av studien?

Resultat från projektet kommer att presenteras på vår hemsida https://www.mom2b.se, i vetenskapliga artiklar och på nationella och internationella konferenser. Studiens resultat

For peer review only

kommer att presentera deltagarnas svar i grupper och dina individuella svar kommer inte att kunna spåras till dig.

Försäkring och ersättning
Som deltagare i denna studie täcks du av patientskadeföreningen. Ingen ersättning kommer att ges för att du deltar i studien.

DELTAGANDET ÄR FRIVILLIGT

Ditt deltagande är frivilligt och du kan när som helst välja att avbryta deltagandet. Om du väljer att inte delta eller vill avbryta ditt deltagande behöver du inte uppge varför, och det kommer inte heller att påverka din framtida vård eller behandling.

Om du vill avbryta ditt deltagande ska du kontakta huvudansvarig forskare, Alkistis Skalkidou (se nedan). Du är också välkommen att kontakta oss på mom2b@kbh.uu.se om du har frågor kring studien.

Ansvarig för studien är:
Professor Alkistis Skalkidou
Institutionen för Kvinnors och Barns hälsa
Akademiska Sjukhuset, 751 85 Uppsala

Epost: mom2b@kbh.uu.se
Telefon: 070 167 97 78

STEG 1: SAMTYCKE
Jag har fått skriftlig information om studien och har haft möjlighet att ställa frågor. Jag har när som helst tillgång till den skriftliga deltagarinformationen elektroniskt i smartphone-applikationen MOM2B.
I de stycken som kommer nu, bestämmer du vilka delar av studien du vill vara med på genom att klicka i rutorna för Ja eller Nej.
Jag godkänner mitt deltagande i studien. (Du måste svara Ja för att kunna vara med i studien.)
□ Ja □ Nej

STEG 2: ENKÄTER
Jag samtycker till att svara på frågor om min hälsa, livsstil och bakgrund samt barnets hälsa.
□ Ja □ Nej

STEG 3: MOBILANVÄNDNING
Jag samtycker till att uppgifter lagras om min allmänna mobil användning. Med detta menas internetförbrukning (kb/s), processoranvändning (CPU%/s), tillgång till internet samt om min telefon är på laddning eller inte.
□ Ja □ Nej

STEG 4: RÖRELSEMÖNSTER

For peer review only

Jag samtycker till att uppgifter lagras om hur mycket jag förflyttar mig. Vi vill registrera hur mycket och hur snabbt du rör dig, men inte exakt var. Bara platsdata från okänd punkt sparas, inte din exakta position. Väljer du "nej" sparas inga platsdata, relativa eller exakta.
□ Ja □ Nej

STEG 5: SOCIALA MEDIER
Jag samtycker till att uppgifter lagras om hur aktiv jag är på sociala medier. Med detta menas antal gånger jag utför en aktivitet (att någon gillar en av dina statusuppdateringar eller att du postar en statusuppdatering) men inte aktivitetens text- eller bildinnehåll. Detta innebär alltså INTE information om vem som kontaktas, vad som skrivs eller vad som gillas.
□ Ja □ Nej

STEG 6: LJUDINSPELNING
Jag samtycker till att spela in ljudet av min röst när jag läser upp förutbestämda texter och siffror.
□ Ja □ Nej

STEG 7: HÄLSOREGISTER
Jag samtycker till att uppgifter hämtas från nationella hälsoregister och vårdkvalitetsregister om min hälsa, livsstil och bakgrund från ett år före till 15 år efter förlossningen. Exempel är diagnoser jag får i kontakt med vården, utbildningsnivå, inkomst och sysselsättning.
□ Ja □ Nej

STEG 8: DELTAGANDE I STUDIER
För att förbättra appen och öka möjligheterna till prediktion av förlossningsdepression samt underlätta för andra studier inom kvinnohälsa, önskar vi fråga dig om vi får kontakta dig vid ett eventuellt senare tillfälle med förfrågan om deltagande i andra studier.
Vid den tidpunkten har du alltid möjlighet att avböja erbjudandet om deltagande.
Deltagande i övriga studier är helt frivilligt och väljer du att inte bli kontaktad kommer du inte att påverkas negativt på något sätt. Markera ett eller båda av följande alternativ om du vill bli kontaktad:
□ Jag önskar bli kontaktad för förfrågan om deltagande i sub-studier inom Mom2B
□ Jag önskar bli kontaktad för förfrågan om deltagande i andra. relevanta studier om kvinnohälsa

UNDERSKRIFT
För att kunna använda appen behöver vi din underskrift
För- och efternamn
Tryck här för att skriva under

For peer review only
